# Dual Ca^2+^-dependent gates in human Bestrophin1 underlie disease-causing mechanisms of gain-of-function mutations

**DOI:** 10.1038/s42003-019-0433-3

**Published:** 2019-06-24

**Authors:** Changyi Ji, Alec Kittredge, Austin Hopiavuori, Nancy Ward, Shoudeng Chen, Yohta Fukuda, Yu Zhang, Tingting Yang

**Affiliations:** 1 Department of Pharmacology and Physiology, University of Rochester, School of Medicine and Dentistry, Rochester, NY 14642 USA; 2 Guangdong Provincial Key Laboratory of Biomedical Imaging, Department of Experimental Medicine, The Fifth Affiliated Hospital of Sun Yat-sen University, 519000 Zhuhai, Guangzhou China; 3 Division of Advance Pharmaco-Science, Graduate School of Pharmaceutical Science, Osaka University, Yamadaoka 1-6, Suita, Osaka 565-0871 Japan

**Keywords:** Chloride channels, Hereditary eye disease, Patch clamp, Permeation and transport, X-ray crystallography

## Abstract

Mutations of human *BEST1*, encoding a Ca^2+^-activated Cl^−^ channel (hBest1), cause macular degenerative disorders. Best1 homolog structures reveal an evolutionarily conserved channel architecture highlighted by two landmark restrictions (named the “neck” and “aperture”, respectively) in the ion conducting pathway, suggesting a unique dual-switch gating mechanism, which, however, has not been characterized well. Using patch clamp and crystallography, we demonstrate that both the neck and aperture in hBest1 are Ca^2+^-dependent gates essential for preventing channel leakage resulting from Ca^2+^-independent, spontaneous gate opening. Importantly, three patient-derived mutations (D203A, I205T and Y236C) lead to Ca^2+^-independent leakage and elevated Ca^2+^-dependent anion currents due to enhanced opening of the gates. Moreover, we identify a network of residues critically involved in gate operation. Together, our results suggest an indispensable role of the neck and aperture of hBest1 for channel gating, and uncover disease-causing mechanisms of hBest1 gain-of-function mutations.

## Abstract (web summary)

Ji and Kittredge et al. demonstrate that the neck and aperture of human Bestrophin1, a Ca^2+^-activated Cl^-^ channel, are both Ca^2+^-dependent gates. By showing hyper-activity of Bestrophin1, which is associated with its enhanced gate opening in the presence of three patient-derived mutations, this study provides mechanistic insights into the gain-of-function mutations that cause macular degenerative disease.

## Introduction

Human bestrophin1 (hBest1) is a Ca^2+^-activated Cl^−^ channel essential for Cl^−^ transport in retinal pigment epithelium (RPE)^1–3^. Over 250 patient-specific mutations in the *BEST1* gene, which encodes hBest1, have been documented to cause retinal degenerative diseases^4–10^, underlying the most direct biomedical relevance known for the channel. Best1 is also distributed in the brain and spinal cord, where its physiological role remains elusive^9^. There are three other human bestrophin paralogs (hBest2–4) that share highly conserved transmembrane regions with hBest1 and were all originally identified as Ca^2+^-activated Cl^−^ channels^11,12^. Therefore, deciphering the structure and function of Best1 has critical biological significance.

Although the structure of hBest1 is still unsolved, two Best1 homolog structures, one from bacteria *Klebsiella pneumoniae* (KpBest) and the other from chicken (cBest1), have been previously reported^13,14^. The KpBest and cBest1 structures are very similar (root-mean-square deviation (RMSD) 2.4 Å) despite the relatively low sequence identity (17%) shared between the two proteins^15^, highlighting the evolutionarily conserved architecture of bestrophins. Overall, the channel is a pentameric assembly with a single ion-conducting pathway, which varies in diameter along its length and is constrained at two narrow points: a “neck” contributed by three residues (I62/I66/F70 in KpBest and I76/F80/F84 in hBest1/cBest1) on a transmembrane helix, and an “aperture” formed by a single residue (I180 in KpBest1, I205 in hBest1, and V205 in cBest1) on a cytosolic helix. The neck and aperture are both apparent candidates for controlling Ca^2+^-dependent activation and ion permeability of the channel, but their regulatory mechanisms and individual contributions to the channel function are still unclear.

The radii of the neck and aperture in the original Ca^2+^-bound cBest1 structure are 1.1–1.3 Å, barely sufficient or insufficient for Cl^−^ even in the dehydrated form (1.8 Å, compared to 3.3 Å in the hydrated form) to pass through. As bigger anions such as Br^−^, I^−^, and SCN^−^ (radius 2.0, 2.2, and 2.2 Å, respectively, in the dehydrated form) are also permeable to cBest1^16^, the cBest1 structure, like the KpBest counterpart, is in a closed or inactivated state. Consistent with this idea, cBest1 structures containing an opened neck are recently obtained by removing a conserved inhibitory sequence at the C-terminus^17,18^. However, the open state conformation of the aperture still remains to be defined.

Gain-of-function mutations displaying elevated channel activity, best exemplified by alanine substitutions at the neck and aperture, have been a critical tool to study channel activation. As both restrictions are formed by side chains of the contributing hydrophobic residues, substitution with a shorter side-chained alanine will enlarge the size of the mutated restriction, resembling channel opening/activating. For instance, the structure of a cBest1 mutant with triple alanine substitution of the neck residues shows a much bigger opening at the neck^16^, such that the neck is no longer a constraint for the passing ions and the aperture represents the only restriction in the ion-conducting pathway, functionally mimicking a state where the neck has been constantly opened/activated. With the same idea, alanine substitution at the aperture functionally mimics constant opening/activation of the aperture, leaving the neck as the sole constraint^14,16^.

In KpBest and hBest1, alanine substitution at the aperture (KpBest I180A and hBest1 I205A) significantly enhanced channel activity^14^, suggesting an activation gate role for the aperture. In cBest1, triple alanine substitution of all three conserved hydrophobic residues at the neck (cBest1 I76A/F80A/F84A, 3A) eliminated Ca^2+^ dependence without altering anion selectivity, while alanine substitution at the aperture (cBest1 V205A) abolished anion selectivity without affecting Ca^2+^ dependence^16^, suggesting an exclusive Ca^2+^-dependent activation gate role for the neck and a Ca^2+^-independent ion filter role for the aperture. These seemingly confusing results could be due to the different Best1 homologs and experimental methods involved in previous studies. In particular, the aperture-forming residue is a long side-chained isoleucine in KpBest (I180) and hBest1 (I205) but a less bulky valine in cBest1 (V205), resulting in a smaller aperture in KpBest/hBest1, which suggests a stronger gating capacity/effect. Consistent with this notion, KpBest I180 is the narrowest point in the channel ion-conducting pathway, but cBest1 V205 is not^13,14^. In Coot simulation, substituting cBest1 V205 with an isoleucine makes it the smallest restriction on cBest1, with the same radius (0.9 Å) as that at KpBest I180, predicting that I205 likely forms the narrowest restriction on hBest1 in the closed/inactivated state. Nevertheless, a careful examination of the neck and aperture in hBest1 is in urgent need.

Here, by electrophysiological and structural analyses, we show that the neck and aperture in hBest1 are both indispensable Ca^2+^-dependent gates, and their dysregulated opening by rationally designed or patient-derived mutations causes Ca^2+^-independent leak and elevates Ca^2+^-dependent channel activity. This illustrates, for the first time to our knowledge, gain-of-function disease-causing mechanisms of *BEST1* mutations. Moreover, we identify a network of critical residues involved in operating the gates, a promotive effect of methanesulfonate^−^ (CH_3_SO_3_^−^) on channel opening through the neck, and an estimated size of the opened aperture. Taken together, our results reveal fundamental gating mechanisms of bestrophin channels with direct pathological relevance.

## Results

### Ca^2+^ dependence of the gates

**Fig. 1:**
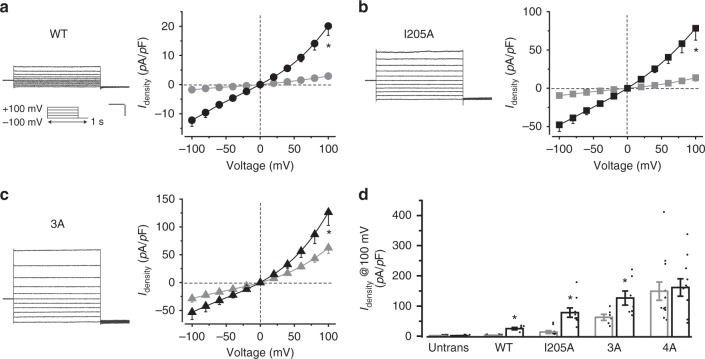
Ca^2+^-dependent Cl^−^ currents in human bestrophin1 (hBest1) gate mutants. **a**–**c** Representative current traces in the presence of 1.2 μM Ca^2+^, and population steady-state current density–voltage relationships in HEK293 cells individually expressing hBest1 wild-type (WT) (**a**), I205A (**b**), or 3A (**c**), with Cl^−^ in the external solution, in the absence (gray) or presence (black) of 1.2 μM Ca^2+^, *n* = 5–10 for each point. Insert, voltage protocol used to elicit currents. Scale bar, 300 pA, 150 ms. *P < 0.05 compared to cells in the absence of Ca^2+^, using two-tailed unpaired Student’s *t* test. **d** Bar chart showing the steady-state Cl^−^ current densities at 100 mV in the absence (gray) or presence (black) of Ca^2+^, *n* = 5–11 for each bar. *P < 0.05 compared to cells in the absence of Ca^2+^, using two-tailed unpaired Student’s *t* test. All error bars in this figure represent s.e.m.

To investigate how the two restrictions in hBest1 cooperate to control channel gating, we performed alanine substitution for the residue(s) at the aperture (I205A), the neck (I76A/F80A/F84A, 3A), and both (I76A/F80A/F84A/I205A, 4A) (Supplementary Fig. 1). As alanine has a much smaller side chain compared to isoleucine or phenylalanine, the substitution is predicted to abolish channel gating at the corresponding restriction by functionally mimicking a constantly open state, allowing us to separate the function of the two restrictions: channel gating in the I205A and 3A mutants relies on the neck and aperture, respectively. When individually transfected into HEK293 cells, wild-type (WT) hBest1 and all the mutants showed similar protein levels in both whole-cell lysate and the membrane fraction (Supplementary Fig. 2), suggesting that neither overall expression nor membrane trafficking of hBest1 is affected by the mutations. Remarkably, without Ca^2+^, all three mutants conducted robust Cl^−^ current in patch clamp recordings, in sharp contrast to the tiny current in cells transfected with WT hBest1, which was indistinguishable from that of untransfected cells (Fig. 1a–d, Supplementary Fig. 3a). This suggests that both the neck and aperture have Ca^2+^-independent spontaneous opening, and neither is dispensable for preventing Ca^2+^-independent leak. Moreover, 1.2 μM Ca^2+^ significantly stimulated Cl^−^ current from I205A and 3A but not that from 4A (Fig. 1b–d, Supplementary Fig. 3a), suggesting that both the neck and aperture are Ca^2+^-dependent gates.

### Opening capacity of the gates

**Fig. 2:**
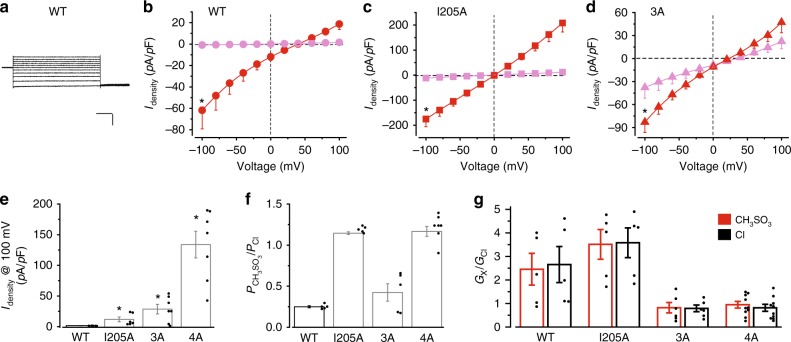
Ca^2+^-dependent CH_3_SO_3_^−^ currents in human bestrophin1 (hBest1) gate mutants. **a** Representative wild-type (WT) current traces in the presence of 1.2 μM Ca^2+^, with CH_3_SO_3_^−^ in the external solution. Scale bar, 200 pA, 150 ms. **b**–**d** Population steady-state current density–voltage relationships in HEK293 cells expressing hBest1 WT (**b**), I205A (**c**), or 3A (**d**), with CH_3_SO_3_^−^ in the external solution, in the absence (magenta) or presence (red) of 1.2 μM Ca^2+^, *n* = 5–6 for each point. *P < 0.05 compared to cells in the absence of Ca^2+^, using two-tailed unpaired Student’s *t* test. **e** Bar chart showing the steady-state CH_3_SO_3_^−^ current densities at 100 mV in the absence of Ca^2+^, *n* = 5–7 for each bar. *P < 0.05 compared to currents conducted by WT hBest1, using two-tailed unpaired Student’s *t* test. **f** Relative ion permeability ratios (*P*_CH3SO3_/*P*_Cl_) calculated from the Goldman–Hodgkin–Katz equation, *n* = 5–7 for each bar. **g** Relative ion conductance ratios (*G*_X_/*G*_Cl_) measured as slope conductance at the reversal potential plus 50 mV (CH_3_SO_3_^−^/Cl^−^, red) or minus 50 mV (Cl^−^/Cl^−^, black), *n* = 5–10 for each bar. All error bars in this figure represent s.e.m.

It was proposed that the aperture in cBest1 (radius 1.3 Å) has “breathing” to allow the passage of dehydrated anions such as Cl^−^, Br^−^, and I^−^ (radius 1.8, 2.0, and 2.2 Å, respectively)^13^. This mechanism is possible, although unproven, considering the resolution of the structure and moderate oversize of the anions. However, the presence of a bulkier isoleucine at the hBest1 aperture (I205) poses a challenge to this hypothesis. To probe the hBest1 pore-opening capacity, we replaced Cl^−^ in the external solution with CH_3_SO_3_^−^, which is an ideal choice for probing Ca^2+^-activated Cl^−^ channels because of its spherical shape, big size (radius 2.6 Å in the dehydrated form), low relative permeability, non-association with Ca^2+^, and complete dissociation at physiological pH^19^. Strikingly, hBest1 conducted robust outward CH_3_SO_3_^−^ current (CH_3_SO_3_^−^ influx) in the presence of 1.2 μM Ca^2+^ (Fig. 2a, b, Supplementary Fig. 3b), indicating that the radius of the opened channel pore is at least 2.6 Å. As the closed neck and aperture in hBest1 presumably have similar sizes to those in cBest1 and KpBest (much smaller than 2.6 Å, Table 1), we concluded that both restrictions must be considerably dilated during channel activation.

**Table 1:** Radii at critical residues in the ion-conducting pathway of bestrophins

KpBest		cBest1	
Residue	Radius (Å)	Residue	Radius (Å)
I62	1.3	I76	1.1
I66	2.2	F80	1.4
F70	2.3	F84	2.3
I180	0.9	V205I	0.9
I180A	3.2	V205	1.3

Radius was calculated based on measuring the distances between five CH_3_- (C delta on isoleucine or valine, a1–a5) or H- (benzene ring on phenylalanine, b1–b5):

For isoleucine or valine, radius = (a1 + a2 + a3 + a4 + a5)/(5 × 1.17) – 2.00 (van der Waals radius of CH_3_-)

For phenylalanine, radius = (b1 + b2 + b3 + b4 + b5)/(5 × 1.17) – 1.20 (van der Waals radius of H-)

### Ion selectivity through the aperture

Consistently, hBest1 I205A, 3A, and 4A mutants all conducted robust CH_3_SO_3_^−^ current in the absence of Ca^2+^ (Fig. 2c–e, Supplementary Fig. 3a), reaffirming spontaneous openings of both restrictions and the minimum radius of 2.6 Å at opening; CH_3_SO_3_^−^ currents from I205A and 3A, but not from 4A, were significantly stimulated by 1.2 μM Ca^2+^ (Fig. 2c, d, Supplementary Fig. 3a), reaffirming that both the neck and aperture are Ca^2+^-dependent gates. The reversal potential of CH_3_SO_3_^−^ in hBest1 was right shifted (Fig. 2b, Supplementary Fig. 3b), indicating that CH_3_SO_3_^−^ is less permeable than Cl^−^. hBest1 WT, I205A, 3A, and 4A displayed a CH_3_SO_3_^−^ to Cl^−^ relative permeability of 0.25 ± 0.01, 1.15 ± 0.02, 0.42 ± 0.11, and 1.17 ± 0.06, respectively (Fig. 2f, Supplementary Fig. 3c), suggesting a major role of the aperture in hBest1 ion selectivity.

### *Trans* promotive effect of CH_3_SO_3_^−^ through the neck

Interestingly, replacing Cl^−^ in the external solution with CH_3_SO_3_^−^ resulted in a strong increase of not only outward current (CH_3_SO_3_^−^ influx) but also inward current (Cl^−^ efflux) in WT hBest1 (Fig. 2b, g, Supplementary Fig. 3b), indicating that CH_3_SO_3_^−^ on the external side of the channel promotes Cl^−^ outward movement from the intracellular side in *trans*. This “*trans* effect” has been documented for Ca^2+^-activated Cl^−^ channels^20,21^ and is suggested to reflect the influence of the ion, CH_3_SO_3_^−^ in this case, on channel gating. Importantly, the *trans* effect was abolished in the 3A and 4A mutants but retained in the I205A mutant (Fig. 2g), suggesting that CH_3_SO_3_^−^ promotes hBest1 gating primarily through the neck.

### Incomplete closure of the aperture is linked to disease

**Fig. 3:**
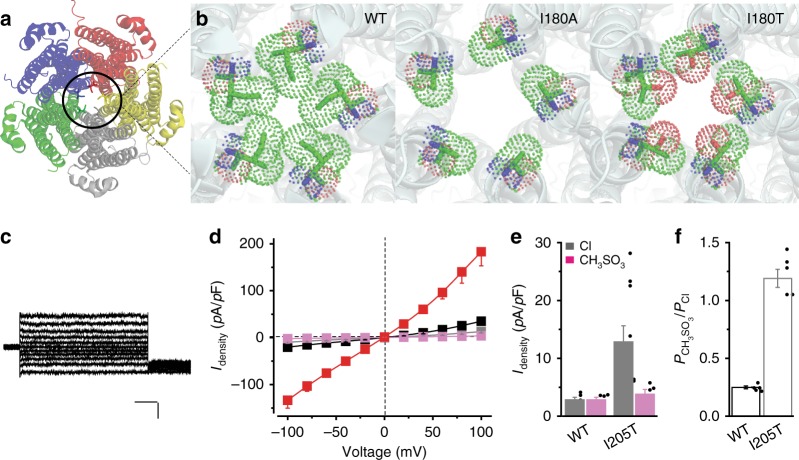
Structural and functional analyses of two aperture mutants. **a** KpBest pentamer viewed from the cytoplasmic side and vertical to the ion-conducting pathway. **b** Apertures of KpBest wild-type (WT), I180A, and I180T as viewed from the same direction as in **a**. Figures were made from actual crystal structures, and critical residues on the apertures are colored by element. **c** Representative human bestrophin1 (hBest1) I205T current traces in the absence of Ca^2+^, with Cl^−^ in the external solution. Scale bar, 100 pA, 150 ms. **d** Population steady-state current density–voltage relationships in HEK293 cells expressing hBest1 I205T, with Cl^−^ in the external solution in the absence (gray) or presence (black) of 1.2 μM Ca^2+^ or with CH_3_SO_3_^−^ in the external solution in the absence (magenta) or presence (red) of 1.2 μM Ca^2+^, *n* = 10–11 for each point. **e** Bar chart showing the steady-state Cl^−^ and CH_3_SO_3_^−^ current densities of hBest1 WT and I205T at 100 mV in the absence of Ca^2+^, *n* = 5–10 for each bar. **f** Relative ion permeability ratio (*P*_CH3SO3_/*P*_Cl_) in hBest1 WT and I205T, *n* = 5–10 for each bar. All error bars in this figure represent s.e.m.

Consistent with the Cl^−^/CH_3_SO_3_^−^ leakage in the hBest1 3A mutant, the equivalent cBest1 3A mutant shows an unconstrained neck (radius 3.2 Å)^16^. To characterize how alanine substitution affects the size of the aperture, we solved the crystal structure of the KpBest I180A mutant (equivalent to hBest1 I205A) at 2.9 Å (Fig. 3a, b, Supplementary Fig. 4a, Table 2, Supplementary Tables 1 and 2). Compared to WT KpBest, the I180A mutant displays a much bigger opening at the aperture (radius 0.9 vs. 3.2 Å) without altering the overall structure of the channel (RMSD = 0.4 Å) (Fig. 3b, Supplementary Table 1). Notably, the structure of this region is well preserved among species (Supplementary Fig. 1), providing a molecular basis for dehydrated Cl^−^ and CH_3_SO_3_^−^ (radius 1.8 and 2.6 Å, respectively) to pass through the aperture of hBest1 I205A in the absence of Ca^2+^.

**Table 2:** Data collection and refinement statistics

	W16A (6IVR)	G18A (6IVJ)	S19A (6IVQ)	P143A (6IVM)
*Data collection*				
Space group	*P* 2_1_ 2_1_ 2_1_	*P* 2_1_ 2_1_ 2_1_	*P* 2_1_ 2_1_ 2_1_	*P* 2_1_ 2_1_ 2_1_
Cell dimensions				
* a*, *b*, *c* (Å)	113.91, 159.57, 161.72	114.12, 160.03, 161.78	113.70, 159.64, 161.30	113.97, 158.81, 161.68
* α*, *β*, *γ* (°)	90, 90, 90	90, 90, 90	90, 90, 90	90, 90, 90
Resolution (Å)	161.7–2.80 (2.86–2.80)^a^	161.78–2.77 (2.82–2.77)^a^	159.6–2.65 (2.70–2.65)^a^	158.82–2.95 (3.03–2.95)^a^
*R*_merge_ (%)	9.2 (136.7)	10.2 (231.0)	6.4 (104.5)	8.7 (90.7)
*I/σ*(*I*)	14.1 (1.4)	15.5 (0.8)	19.1 (2.0)	11.1 (1.7)
CC_1/2_	(0.524)	(0.333)	(0.662)	(0.666)
Completeness (%)	99.9 (99.9)	99.5 (95.5)	99.9 (100)	97.7 (98.8)
Redundancy	6.7 (6.5)	6.7 (5.9)	6.8 (6.9)	3.4 (3.5)
*Refinement*				
Resolution (Å)	48.7–2.80 (2.87–2.80)	48.8–2.77 (2.84–2.77)	48.6–2.65 (2.72–2.65)	48.7–2.95 (3.03–2.95)
No. of reflections	69,361	71,525	81,334	57,824
*R*_work_/*R*_free_	20.9/24.4	21.5/24.4	20.1/23.9	20.8/24.3
No. of atoms				
Protein	10,726	10,708	10,764	10,754
Ligand/Zn	38	31	55	55
Water	63	69	79	79
*B* factors	77.1	82.7	75.0	80.9
Protein	77.1	82.7	75.2	81.0
Ligand/Zn	73.7	85.4	78.2	85.2
Water	67.1	76.6	63.0	62.2
R.m.s. deviations				
Bond lengths (Å)	0.009	0.011	0.005	0.009
Bond angles (°)	1.06	1.61	1.410	1.406

	G175A (6IVK)	D179A (6JLF)	I180A (6IV0)	I180T (6IV1)
*Data collection*				
Space group	*P* 2_1_ 2_1_ 2_1_	*P* 2_1_ 2_1_ 2_1_	*P* 2_1_ 2_1_ 2_1_	*P* 2_1_ 2_1_ 2_1_
Cell dimensions				
* a*, *b*, *c* (Å)	113.55, 159.42, 161.48	114.0, 159.5, 161.9	113.6, 159.9, 161.3	112.8, 159.2, 159.9
* α*, *β*, *γ* (°)	90, 90, 90	90, 90, 90	90, 90, 90	90, 90, 90
Resolution (Å)	161.5–2.65 (2.70–2.65)^a^	93.19–2.55 (2.64–2.55)^a^	60.81–2.9 (3.0–2.9)^a^	112.84–3.18 (3.29–3.18)^a^
*R*_merge_ (%)	6.4 (85.9)	52 (120.4)	3.4 (45.9)	8.5 (95)
*I/σ*(*I*)	20.1 (2.3)	18.74 (1.17)	17.63 (1.69)	8.18 (0.95)
CC_1/2_	(0.765)	(0.447)	(0.657)	(0.338)
Completeness (%)	100 (100)	98.2 (97.2)	99.8 (99.9)	87.2 (88.2)
Redundancy	6.8 (6.7)	3.4 (3.5)	2.0 (2.0)	1.9 (1.8)
*Refinement*				
Resolution (Å)	48.6–2.65 (2.72–2.65)	93.19–2.55 (2.64–2.55)	60.81–2.9 (3.0–2.9)	112.84–3.18 (3.29–3.18)
No. of reflections	81,356	95,113	65,642	42,861
*R*_work_/*R*_free_	20.1/24.1	23.3/28.1	20.6/23.9	22.9/26.6
No. of atoms				
Protein	10,751	10,477	10,621	10,570
Ligand/Zn	60	11	15	15
Water	124	53	24	8
*B* factors	71.7	84.15	81.27	96.24
Protein	71.8	84.23	81.33	96.27
Ligand/Zn	77.2	87.8	85.78	106.42
Water	63.0	67.46	54.33	38.02
R.m.s. deviations				
Bond lengths (Å)	0.005	0.011	0.01	0.009
Bond angles (°)	1.387	1.2	1.4	1.3

	P208A (6IVO)	Y211A (6IV2)	W252A (6IV3)	W252F (6IV4)
*Data collection*				
Space group	*P* 2_1_ 2_1_ 2_1_	*P* 2_1_ 2_1_ 2_1_	*P* 2_1_ 2_1_ 2_1_	*P* 2_1_ 2_1_ 2_1_
Cell dimensions				
* a*, *b*, *c* (Å)	114.01, 160.06, 161.84	114.16, 160.11, 162.04	113.46, 159.61, 162.67	104.01, 154.19, 161.74
* α*, *β*, *γ* (°)	90, 90, 90	90, 90, 90	90, 90, 90	90, 90, 90
Resolution (Å)	160.1–2.45 (2.49–2.45)^a^	71.77–2.62 (2.71–2.62)^a^	66.11–2.52 (2.61–2.52)^a^	61.93–3.14 (3.25–3.14)^a^
*R*_merge_ (%)	6.7 (97.7)	5.7 (114)	6.7 (95.2)	9.1 (110.6)
*I/σ*(*I*)	17.7 (2.2)	14.28 (1.10)	12.75 (1.36)	8.77 (1.04)
CC_1/2_	(0.755)	(0.275)	(0.491)	(0.359)
Completeness (%)	100 (100)	97.2 (98.2)	98.7 (95.9)	98.5 (97.4)
Redundancy	6.8 (6.9)	3.5 (3.3)	3.4 (3.4)	3.4 (3.2)
*Refinement*				
Resolution (Å)	48.8–2.45 (2.51–2.45)	71.77–2.62 (2.71–2.62)	66.11–2.52 (2.61–2.52)	61.93–3.14 (3.25–3.14)
No. of reflections	103,810	87,896	99,774	45,665
*R*_work_/*R*_free_	20.2/23.8	23.0/26.1	23.6/26.2	24.7/29.5
No. of atoms				
Protein	10,769	10,503	10,692	10,491
Ligand/Zn	94	12	15	13
Water	202	36	80	0
*B* factors	64.1	91.97	68.28	97.81
Protein	64.1	92.05	68.33	97.82
Ligand/Zn	75.6	93.57	68.69	96.07
Water	61.8	67.01	61.38	—
R.m.s. deviations				
Bond lengths (Å)	0.005	0.007	0.01	0.011
Bond angles (°)	1.288	1.09	1.16	1.34

	L259A (6IVL)	P262A (6IVP)	G264A (6IVN)	D269A (6IVW)
*Data collection*				
Space group	*P* 2_1_ 2_1_ 2_1_	*P* 2_1_ 2_1_ 2_1_	*P* 2_1_ 2_1_ 2_1_	*P* 2_1_ 2_1_ 2_1_
Cell dimensions				
* a*, *b*, *c* (Å)	114.35, 160.54, 161.88	114.48, 162.41, 161.79	114.15, 160.94, 160.96	113.96, 161.77, 162.37
* α*, *β*, *γ* (°)	90, 90, 90	90, 90, 90	90, 90, 90	90, 90, 90
Resolution (Å)	161.9–3.40 (3.54–3.40)^a^	162.4–3.80 (4.03–3.80)^a^	160.9–3.10 (3.20–3.10)^a^	162.37–3.72 (3.98–3.72)^a^
*R*_merge_ (%)	8.8 (83.5)	9.0 (88.7)	5.8 (73.0)	21 (100.6)
*I/σ*(*I*)	15.4 (2.6)	7.7 (1.8)	13.2 (1.8)	5.5 (1.3)
CC_1/2_	(0.757)	(0.506)	(0.610)	(0.421)
Completeness (%)	100 (100)	98.2 (99.1)	98.4 (99.5)	82.4 (85.2)
Redundancy	6.6 (6.8)	3.3 (3.3)	3.4 (3.4)	3.9 (4.0)
*Refinement*				
Resolution (Å)	48.8–3.40 (3.49–3.40)	50.1–3.80 (3.90–3.80)	48.6–3.10 (3.18–3.10)	81.2–3.72 (3.81–3.72)
No. of reflections	39,546	28,119	50,638	26,197
*R*_work_/*R*_free_	20.9/24.4	26.6/31.5	21.3/26.5	29.9/36.1
No. of atoms				
Protein	10,699	10,744	10,770	10,533
Ligand/Zn	26	24	26	10
Water	10	1	24	0
*B* factors	104.0	159.6	98.4	112.8
Protein	104.0	159.8	98.5	112.8
Ligand/Zn	104.1	150.2	103.4	112.2
Water	83.9	103.2	72.7	—
R.m.s. deviations				
Bond lengths (Å)	0.012	0.007	0.003	0.003
Bond angles (°)	1.36	1.161	0.753	0.635

^a^Values in parentheses are for highest-resolution shell

A patient-derived mutation of hBest1 at the aperture, I205T, was previously reported^6^. As threonine has a shorter side chain than isoleucine, we speculated that I205T, like I205A, enlarges the opening at the aperture and enables Ca^2+^-independent leak activity. Notably, the threonine side chain possesses a hydrophilic hydroxyl group that is capable of forming H-bonds, which can further affect ion permeation. To test this idea, we solved the crystal structure of the equivalent KpBest I180T mutant at 3.2 Å (Supplementary Fig. 4a, Table 2, Supplementary Tables 1 and 2) and examined hBest1 I205T in HEK293 cells by patch clamp. Remarkably, the aperture of KpBest I180T was dilated to a size (radius 2.1 Å) bigger than dehydrated Cl^−^ but smaller than CH_3_SO_3_^−^ (Fig. 3b), while hBest1 I205T conducted robust Cl^−^ current but no CH_3_SO_3_^−^ current in the absence of Ca^2+^ (Fig. 3c–e, Supplementary Fig. 3d). Moreover, hBest1 I205T showed a CH_3_SO_3_^−^ to Cl^−^ relative permeability similar to that of hBest1 I205A (Fig. 3f) and retained CH_3_SO_3_^−^-mediated *trans* effect (Fig. 3d, Supplementary Fig. 3d), reaffirming the critical roles of the aperture and the neck in ion selectivity and CH_3_SO_3_^−^-promoted channel opening, respectively. Therefore, our results indicate that hBest1 I205T is a gain-of-function mutation due to incomplete closure of the aperture.

Interestingly, the Cl^−^ and CH_3_SO_3_^−^ currents conducted by I205V are similar to those from WT hBest1 (Supplementary Fig. 3e), suggesting that swapping the cBest1 aperture onto hBest1 does not significantly affect the function of hBest1.

### Structure–function analysis of highly conserved residues

To further dissect the mechanism of hBest1 activation, we carried out an alanine substitution screen for mutations with altered opening of the neck and/or aperture. Twelve identical residues shared between KpBest and hBest1 on the loop structures within the channel core transmembrane regions were identified as the subjects of alanine substitution (Supplementary Fig. 5 and Supplementary Table 1), as we reasoned that replacing conserved residues on the (relatively flexible) loops, rather than substitution on the rigid helices, are more likely to be informative without disrupting the overall channel structure. Importantly, these 12 residues are hotspots of disease-causing mutations: 10 of them have been identified as mutation sites in patients, while 4 of them are associated with multiple types of mutations. This provides a validation of our reasoning.

Alanine substitution mutants for each of the 12 residues were generated in KpBest and hBest1 and subjected to crystallography and patch clamp, respectively. All 12 KpBest mutants were purified with a size exclusion profile indistinguishable from the WT (Supplementary Fig. 6), suggesting that the overall integrity of the channel is still retained after alanine substitution. Eleven of the 12 KpBest mutant structures were solved (Table 2, Supplementary Tables 1 and 2), while 10 of the 12 hBest1 mutants displayed altered channel function (Supplementary Fig. 7). Several mutations are particularly interesting.

### hBest1 P233A: loss-of-function likely influencing the neck

**Fig. 4:**
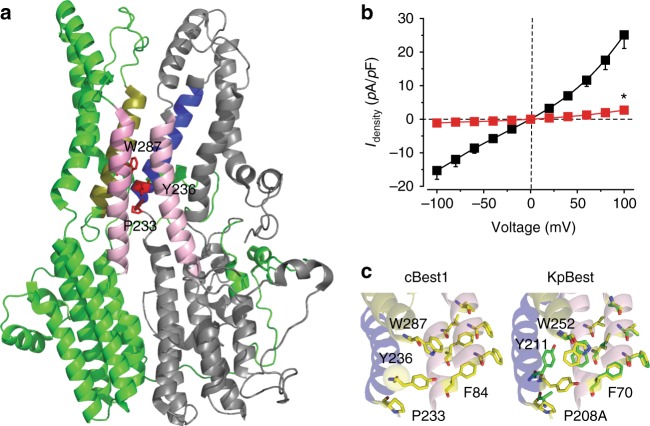
Structural and functional analyses of human bestrophin1 (hBest1) P233A/KpBest P208A. **a** Ribbon diagram of two adjacent (72°) protomers of a cBest1 pentamer with the extracellular side on the top. The side chains of P233, Y236, and W287 are shown in red. Helices surrounding the critical residues (F84, P233, Y236, and W287) are labeled in the same colors as those in **c** for comparison. **b** Population steady-state current density–voltage relationships in HEK293 cells expressing hBest1 WT (black) and P233A (red), with Cl^−^ in the external solution in the presence of 1.2 μM Ca^2+^, *n* = 5–6 for each point. *P < 0.05 compared to cells expressing WT hBest1, using two-tailed unpaired Student’s *t* test. **c** Visualization of the structural alteration. cBest1, Ca^2+^ labeled as yellow sphere; KpBest, showing critical residues on WT (yellow) and P208A (green). All error bars in this figure represent s.e.m.

hBest1 P233A is a patient-derived mutation localized on the C-terminus of a loop connecting to helix S3b (Fig. 4a, Supplementary Fig. 5). Tiny Cl^−^ current indistinguishable from that of the untransfected control was recorded in HEK293 cells expressing the hBest1 P233A mutant (Fig. 4b), despite normal expression and membrane trafficking (Supplementary Fig. 2), indicating severely impaired channel function. The corresponding KpBest P208A showed alterations in the local structure (2.5 Å resolution), as the hydrogen bond network around P208 is changed by the alanine substitution. Consequently, the conformations of Y211 and W252 (equivalent to Y236 and W287 in hBest1/cBest1) located near F70 (equivalent to F84 in hBest1/cBest1), which is one of the three neck-forming residues, are both changed (Fig. 4c, Supplementary Fig. 4b). Y236 is also very close to the bound Ca^2+^ on the Ca^2+^ clasp (Fig. 4c). Notably, the location and position of these critical residues within the channel structure are well preserved among species (Fig. 4c), and patient mutations have been reported on all involved residues in hBest1 (F84, P233, Y236, and W287). Therefore, the P233A mutation may impair opening of the neck through Y236 and W287.

### hBest1 Y236 and W287: critical for gating at the neck

**Fig. 5:**
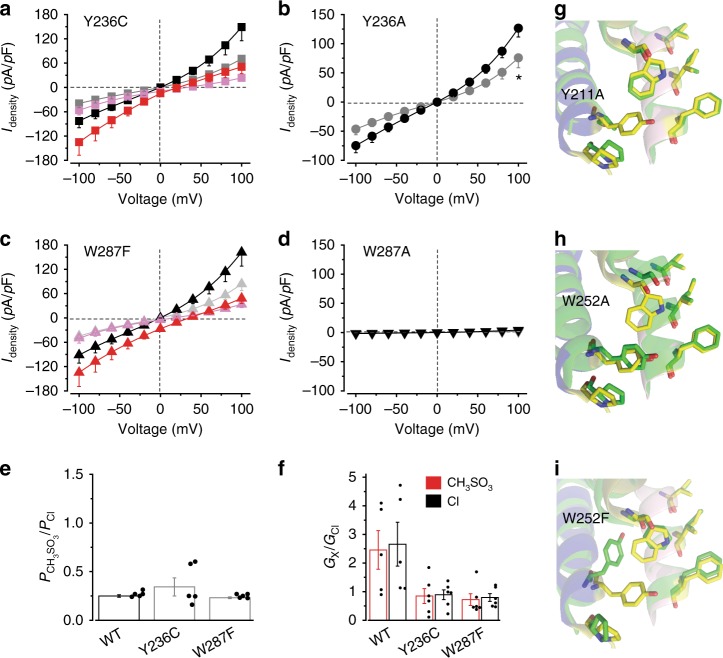
Structural and functional analyses of human bestrophin1 (hBest1) Y236 and W287. **a** Population steady-state current density–voltage relationships in HEK293 cells expressing hBest1 Y236C with Cl^−^ in the external solution in the absence (gray) or presence (black) of 1.2 μM Ca^2+^ or with CH_3_SO_3_^−^ in the external solution in the absence (magenta) or presence (red) of 1.2 μM Ca^2+^, *n* = 5–6 for each point. **b** Population steady-state current density–voltage relationships in HEK293 cells expressing hBest1 Y236A with Cl^−^ in the external solution in the absence (gray) or presence (black) of 1.2 μM Ca^2+^, *n* = 7–8 for each point. *P < 0.05 compared to cells in the presence of Ca^2+^, using two-tailed unpaired Student’s *t* test. **c** Results from hBest1 W287F in the same format as **a**, *n* = 8–11 for each point. **d** Results from W287A in same format as **b**, *n* = 5 for each point. **e** Relative ion permeability ratios (*P*_CH3SO3_/*P*_Cl_) calculated from the Goldman–Hodgkin–Katz equation, *n* = 5 for each bar. **f** Relative ion conductance ratios (*G*_X_/*G*_Cl_) measured as slope conductance at the reversal potential plus 50 mV (CH_3_SO_3_^−^/Cl^−^, red) or minus 50 mV (Cl^−^/Cl^−^, black), *n* = 5–6 for each bar. **g**–**i** Visualization of the neck regions of KpBest Y211A (**g**), W252A (**h**), and W252F (**i**), showing critical residues on WT (yellow) and the mutants (green). Helices surrounding the critical residues (F70, P208, Y211, and W252) are labeled in the same colors as those in Fig. 4 for comparison. All error bars in this figure represent s.e.m.

To further investigate Y236 and W287 on hBest1, we examined two mutants of each residue, including their individual alanine substitution mutants (Y236A and W287A), Y236C (a patient-derived mutation), and W287F (a milder alteration compared to W287A), all of which showed normal overall expression and membrane trafficking in HEK293 cells after transient transfection (Supplementary Fig. 2). The Y236A, Y236C, and W287F mutants all conducted Ca^2+^-dependent Cl^−^ currents significantly bigger than those from WT hBest1 under the same conditions (Fig. 5a–c, Supplementary Fig. 3f), indicating gain-of-function phenotypes. Moreover, the Y236C and W287F mutants retained the CH_3_SO_3_^−^ to Cl^−^ relative permeability but not the *trans*-promotive effect of CH_3_SO_3_^−^ (Fig. 5e, f, Supplementary Fig. 3c), functionally resembling hBest1 3A, rather than hBest1 I205A. As the cBest1 3A mutant structure shows a constantly unconstrained neck^16^, the gain-of-function in Y236A, Y236C, and W287F is likely a result of enhanced open probability of the neck. Notably, Ca^2+^ does not make any significant difference in CH_3_SO_3_^−^ to Cl^−^ relative permeability in 3A, Y236C, or W287F (Supplementary Fig. 3c).

By sharp contrast, cells expressing hBest1 W287A conducted tiny current just like untransfected cells (Fig. 5d), suggesting that, to the opposite of W287F, W287A is a loss-of-function mutation. Therefore, W287 is an extremely sensitive “switch” of the neck.

To understand the structural bases of these mutants, we solved the structures of KpBest Y211A, W252A, and W252F at 2.6, 2.5, and 3.1 Å (equivalent to hBest1 Y236A, W287A, and W287F), respectively. Consistent with the functional results, no structural change was found at the aperture. Although the structure at the neck region was surprisingly unaltered, possibly due to the molecular packing effect in the crystal or the presence of Zn^2+^ in the crystallization solution which might stabilize the channel in a closed state, all three KpBest mutants displayed conformational changes on P208, Y211, and W252 (equivalent to P233, Y236, and W287 in hBest1, respectively) (Fig. 5g–i, Supplementary Fig. 4b), reaffirming the mutual influence between these three residues. Taken together, we concluded that hBest1 P233, Y236, and W287 are critical residues involved in channel gating at the neck.

### hBest1 D203A: gain-of-function influencing both gates

**Fig. 6:**
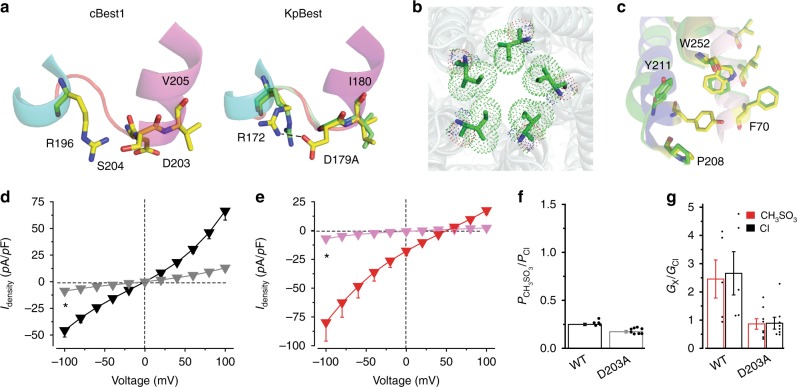
Structural and functional analyses of human bestrophin1 (hBest1) D203A/KpBest D179A. **a** Visualization of the structural alteration, showing critical residues on wild-type (WT; yellow) and the mutant (green). The loop is labeled in red for WT and in green for the KpBest D179A mutant. **b** Aperture of KpBest D179A as viewed from the same direction as in Fig. 3a, b. Critical residues on the apertures are colored by element. **c** Visualization of the neck region of KpBest D179A, showing critical residues on WT (yellow) and the mutant (green). Helices surrounding the critical residues (F70, P208, Y211, and W252) are labeled in the same colors as those in Fig. 4 for comparison. **d** Population steady-state current density–voltage relationships in HEK293 cells expressing hBest1 D203A, with Cl^−^ in the external solution in the absence (gray) or presence (black) of 1.2 μM Ca^2+^, *n* = 12–14. *P < 0.05 compared to cells in the presence of Ca^2+^, using two-tailed unpaired Student’s *t* test. **e** Population steady-state current density–voltage relationships in HEK293 cells expressing hBest1 D203A, with CH_3_SO_3_^−^ in the external solution in the absence (magenta) or presence (red) of 1.2 μM Ca^2+^, *n* = 8–9 for each point. *P < 0.05 compared to cells in the presence of Ca^2+^, using two-tailed unpaired Student’s *t* test. **f** Relative ion permeability ratios (*P*_CH3SO3_/*P*_Cl_) calculated from the Goldman–Hodgkin–Katz equation, *n* = 5–8 for each bar. **g** Relative ion conductance ratios (*G*_X_/*G*_Cl_) measured as slope conductance at the reversal potential plus 50 mV (CH_3_SO_3_^−^/Cl^−^, red) or minus 50 mV (Cl^−^/Cl^−^, black), *n* = 5–8 for each bar. All error bars in this figure represent s.e.m.

hBest1 D203A is a patient-derived mutation localized on an intracellular loop adjacent to the aperture (Fig. 6a, Supplementary Figs. 1 and 5). Structural analysis predicted a local alteration by this alanine substitution, as the corresponding residue in KpBest (D179) is involved in a D179–R172 salt bridge formation (Fig. 6a). Strikingly, the KpBest D179A structure showed a bigger opening at the aperture (radius 1.5 Å) with a slightly twisted I180 possibly caused by disruption of the D179–R172 salt bridge (Fig. 6a, b, Supplementary Fig. 4a), and conformational changes at all three newly identified neck-controlling residues (P208, Y211, and W252; Fig. 6c, Supplementary Fig. 4b). This suggests a crucial role of this residue (KpBest D179 and hBest1 D203) in the regulation of both the neck and aperture. Notably, the aspartic acid–arginine salt bridge is not present in hBest1 because the residue corresponding to R172 of KpBest1 (R196 in cBest1) is W196 in hBest1. However, hBest1 D203 might have interactions with surrounding residues such as P204 through van der Waals contacts in a similar way that cBest1 D203 forms an H-bond with S204 next to the aperture residue (V205) (Fig. 6a).

Consistently, hBest1 D203A displayed electrophysiological properties reflecting changes at both gates: first, the Ca^2+^-dependent Cl^−^ current was bigger than that from WT hBest1, indicating a gain-of-function phenotype (Fig. 6d); second, Ca^2+^-independent leak of Cl^−^, but not of CH_3_SO_3_^−^, was recorded (Fig. 6d, e), suggesting an incomplete closure of the aperture (like hBest1 I205T); third, while the relative permeability of CH_3_SO_3_^−^/Cl^−^ was retained (Fig. 6f), the *trans*-promotive effect of CH_3_SO_3_^−^ was abolished (Fig. 6g), indicating dysregulated gating at the neck (like hBest1 3A, Y236A/C, and W287F). Notably, the D203A mutant behaved similarly to WT hBest1 in terms of protein expression, membrane trafficking, and current rectification (Supplementary Figs. 2 and 3g). Taken together, our results suggest that D203 serves as a communication point of the two gates, while hBest1 D203A is a gain-of-function mutation due to enhanced opening of both the neck and aperture.

### Residues involved in Ca^2+^ binding

**Fig. 7:**
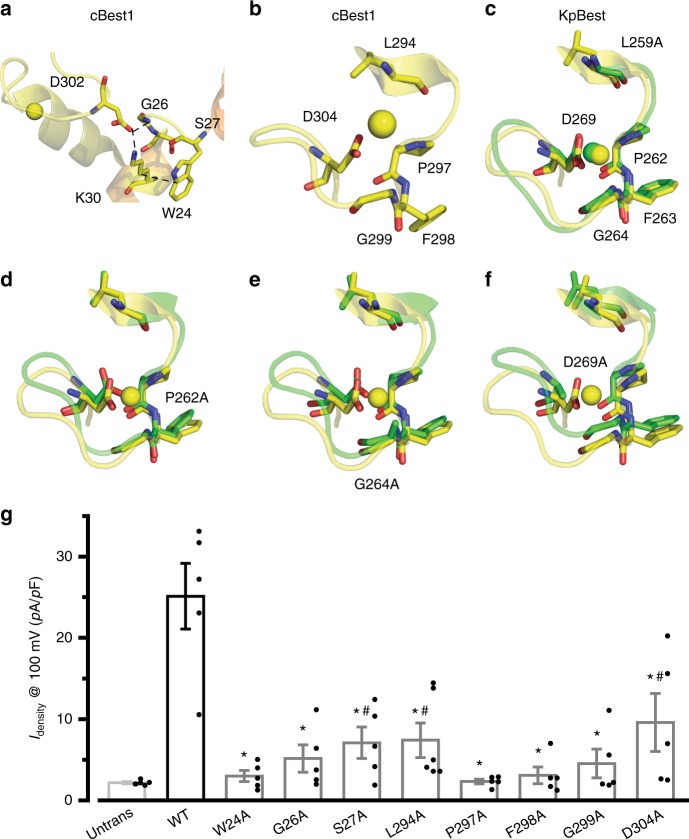
Structural and functional analyses of critical residues on the Ca^2+^ loops. **a**, **b** Visualization of the cBest1 Ca^2+^-binding site composed of an N-terminal loop (**a**) and a Ca^2+^ clasp (**b**). Critical residues are shown with their side chains. Helices surrounding the critical residues are labeled in the same colors as those in Supplementary Fig. 1 for comparison. Yellow sphere, Ca^2+^. **c** Visualization of the region corresponding to the Ca^2+^ clasp in KpBest, which is a Zn^2+^-binding site. Critical regions and residues are labeled yellow in wild-type (WT) and green in the L259A mutant. Zn^2+^ sphere is yellow in WT and green in the L259A mutant. **d**–**f** Visualization of the structural alterations in KpBest P262A (**d**), G264A (**e**), and D269A (**f**). Critical regions and residues are labeled yellow in WT and green in the mutants. Zn^2+^ sphere is yellow in WT. **g** Bar chart showing the population steady-state current densities at 100 mV in HEK293 cells expressing WT and the alanine substitution mutants, *n* = 5-6 for each point. *^,#^*P* < 0.05 compared to currents conducted by WT and untransfected cells, respectively, using two-tailed unpaired Student’s *t* test. All error bars in this figure represent s.e.m.

hBest1 L294, P297, F298, G299, and D304 are localized on an intracellular loop previously identified as the Ca^2+^ clasp in the cBest1 structure^13^ (Fig. 7a–f, Supplementary Figs. 1 and 5), while hBest1 W24, G26, and S27 presumably all communicate with D302 in the Ca^2+^ clasp: G26 forms an H-bond with D302; W24 and S27 interact with K30 through a CH–pi stacking interaction and an H-bond, respectively, and K30 forms a salt bridge with D302 (Fig. 7a). Hence, mutations of these residues likely affect Ca^2+^ binding. Consistently, the eight corresponding hBest1 alanine substitution mutants all displayed significantly smaller currents at 1.2 μM [Ca^2+^]_i_ compared to WT hBest1 in HEK293 cells^22^ (Fig. 7g, Supplementary Fig. 7), indicating defective Ca^2+^-dependent activation. Moreover, the equivalent KpBest mutants (W16A, G18A, S19A, L259A, P262A, G264A, and D269A) all showed an overall similar structure to that of WT KpBest (Supplementary Table 1). Therefore, we concluded that these eight residues are important for channel activation due to their involvement in Ca^2+^ binding.

The corresponding “Ca^2+^-binding” sites in WT KpBest are occupied by Zn^2+^ (Fig. 7c–f), an essential component in the crystallization buffer. Interestingly, the KpBest P262A, G264A, and D269A mutants show no Zn^2+^ in the corresponding sites (Fig. 7d–f). In KpBest G264A, the methyl group of alanine, which is bulkier than glycine, may inhibit the binding of Zn^2+^. Moreover, KpBest L259A, P262A, G264A, and D269A all have relatively low resolution and slightly altered structures in this region (Fig. 7c–f, Table 2), suggesting that the binding of Zn^2+^ stabilizes the channel structure.

## Discussion

A unique feature of Best1 channels, as indicated by the solved KpBest and cBest1 structures, is the presence of two restrictions, the neck and aperture, in the ion-conducting pathway. Given hBest1’s clear association with retinal diseases, it is of obvious importance to understand how these two restrictions on hBest1 contribute to channel gating. Here, by electrophysiological comparison of hBest1 mutants in which either one or both of the restrictions are removed, we demonstrated that the neck and aperture in hBest1 are both Ca^2+^-dependent gates with spontaneous openings in the absence of Ca^2+^, such that the co-existence of two gates is necessary to prevent Ca^2+^-independent leak. By replacing Cl^−^ with CH_3_SO_3_^−^ in the patch clamp external solution, we showed that both restrictions must be considerably dilated (at least to a radius of 2.6 Å) upon channel activation and discovered a CH_3_SO_3_^−^-promoted *trans* effect through the neck. Importantly, using electrophysiological and crystallographic approaches, we identified three patient-derived mutations as gain of function due to enhanced opening at either one or both of the gates, directly linking impaired channel gating to disease-causing mechanisms. Moreover, in an alanine substitution screen, we identified multiple residues (D203, P233, Y236, and W287) critically involved in channel gating at the neck and/or aperture.

While mutants with conformational changes at the aperture (e.g., KpBest I180A, I180T) or neck (e.g., cBest1 3A) do not have structural alteration at the other restriction, a mutation near the aperture (D203A and D179A in hBest1 and KpBest, respectively) impacts both gates, suggesting both independent and cooperative regulations of the two channel gates. Based on these observations, we propose that the neck and aperture in hBest1 serve as two separate Ca^2+^-activated gates communicating through the I205–D203–P233–Y236/W287–F84 network. Notably, hBest1 D203/KpBest D179 is located on an intracellular loop essential for ATP-dependent activation of the channel^15^, potentiating the effect of ATP on both gates.

The Ca^2+^-independent leak current in hBest1 3A was bigger compared to that in hBest1 I205A, suggesting a lower spontaneous open probability of the neck than the aperture, which is consistent with the formation of the neck by three rings of residues as opposed to only one ring of residues at the aperture. In the presence of Ca^2+^, the Cl^−^ currents in hBest1 3A and I205A were enhanced to approximately two- and five-fold of those without Ca^2+^, respectively (compared to approximately nine-fold increase in WT), suggesting that Ca^2+^ has different stimulatory effects on the open probability of the aperture and neck. Consistent with this idea, KpBest I180A (equivalent to hBest1 I205A) displayed dramatically increased open probability but unchanged unitary current in lipid bilayer^14^.

Although bestrophins have been characterized as Ca^2+^-activated Cl^−^ channels, a wide range of Ca^2+^ dependence and sensitivity among them have been documented. For instance, hBest2 and *Drosophila* Best1 (dBest1) expressed in HEK293 cells exhibit Ca^2+^-independent activity^23^, while hBest1 and cBest1 are both Ca^2+^ dependent but display over nine-fold difference in Ca^2+^ sensitivity^16,22,24^. The existence of two gates, both of which allow Ca^2+^-dependent regulation, provides a versatile system for generating distinct Ca^2+^ responses in different bestrophin homologs, which conduct anion currents at various [Ca^2+^]_i_ under physiological conditions. In particular, the aperture-forming residue is variable (e.g., isoleucine, valine, and phenylalanine in hBest1, cBest1, and dBest1, respectively), in sharp contrast to the highly conserved neck-forming residues (identical in hBest1, cBest1, and dBest1), underlining the aperture as a key regulatory element for Ca^2+^ response.

Studies in cBest1 showed that alanine substitution at the aperture (cBest1 V205A) results in a loss of the permeability sequence for anions, suggesting a “size-selective” filter role of the aperture: it represents a constraint to hydrated anions even when opened in WT channels, such that the permeability sequence of different anions is mainly determined by their dehydration energy, while in the mutant channel the opened aperture is sufficient to pass hydrated anions, bypassing the dehydration process and thus resulting in equalized permeability^14,16^. Consistent with this idea, hBest1 I205A and I205T both displayed similar permeability to Cl^−^ and CH_3_SO_3_^−^, which is significantly different in the WT. Importantly, the robust Ca^2+^-dependent CH_3_SO_3_^−^ current in WT hBest1 and CH_3_SO_3_^−^ leak in hBest1 3A indicate that the minimum size of the opened hBest1 aperture should be sufficient to accommodate a dehydrated CH_3_SO_3_^−^ (radius 2.6 Å). On the other hand, to function as a size-selective filter, the opened aperture should not be bigger than hydrated anions (radius 3.3 Å for Cl^−^/Br^−^/I^−^). Therefore, we predict that the hBest1 aperture in its open state has a radius of 2.6–3.3 Å. Interestingly, asymmetry of the aperture is observed in the KpBest D179A and I180T mutants (Figs. 3b and 6b), suggesting that “breathing”, which manifests as local, asymmetric changes in the aperture region, may additionally contribute to accommodate large ions, such as CH_3_SO_3_^−^.

The critical roles of hBest1 Y236 and W287 in gating of the neck have been confirmed by recently solved cBest1 structures with an open state neck^17^. Opening of the cBest1 neck is mediated by a conformational change of the neck-forming S2b helices, which turns the side chains of I76, F80, and F84 away from the central axis of the channel pore. Importantly, F80 and F84 are stabilized in the open conformation by interactions with hydrophobic residues Y236 and W287. Moreover, consistent with our conclusion that hBest1 W287F is a gain-of-function mutation with increased open probability of the neck, a cBest1 W287F mutant structure shows a constantly opened neck^17^. However, the aperture remains unchanged in a presumably closed conformation in all solved cBest1 structures regardless of the status of the neck, possibly reflecting a lower open probability of the aperture and/or the requirement of additional activator(s), such as ATP^15^.

The hBest1 4A mutant is designed to be deficient in gating at both the neck and aperture, because the cBest1 3A and KpBest I180A mutant exhibit an unconstrained neck and aperture for Cl^−^, respectively. As expected, the currents conducted by hBest1 4A in the absence of Ca^2+^ are significantly bigger than those from WT in the presence of Ca^2+^. Moreover, Ca^2+^ has no promotive effect on hBest1 4A, suggesting that the neck and aperture of the 4A mutant either cannot further dilate in response to Ca^2+^ or their further dilation does not contribute to the anion current amplitude (conductance). Interestingly, hBest1 I205A conducted significantly bigger currents compared to hBest1 4A in the presence of Ca^2+^ with CH_3_SO_3_^−^ in the external solution (Supplementary Fig. 3a), suggesting a further dilated neck in hBest1 I205A and thus supporting the first scenario. Consistent with this idea, the size of the fully dilated neck in cBest1 is bigger than that of the cBest1 3A mutant^16,17^. Comparing hBest1 3A to 4A, the former can be stimulated by Ca^2+^ while the latter cannot, suggesting that the Ca^2+^-sensitivity of the 3A mutant is attributed to the aperture.

Here we identified 18 mutations that significantly alter hBest1 channel activity without affecting the whole cell and total membrane expression levels of the protein. Notably, the short extracellular domains of hBest1 represent a challenge for quantitative measurement of the surface expression by labeling techniques (e.g., biotin). They can be classified into three groups by different molecular mechanisms: (1) mutations on residues involved in Ca^2+^ binding, including W24A, G26A, S27A, L294A, P297A, F298A, G299A, and D304A, likely cause defects of Ca^2+^-dependent activation^13^; (2) mutations at the neck/aperture, including I205A, I205T, 3A, and 4A, increase open probability by functionally mimicking a constant open state; and (3) mutations outside of the neck/aperture, including D203A, P233A, Y236A, Y236C, W287A, and W287F, alter open probability through the neck/aperture residues. The latter two groups of mutations may or may not affect channel unitary conductance, which requires further investigation such as single channel recordings.

Previously, only loss-of-function phenotypes have been recognized for *BEST1* patient mutations. To our knowledge, for the first time, we identified gain-of-function disease-causing mutations (D203A, I205T, and Y236C) and revealed the underlying molecular mechanisms. As *BEST1* patients clinically display decreased light peak, which is presumably generated by hBest1-mediated Cl^−^ current in RPE, gain of function represents a disease-causing mechanism fundamentally distinct from loss of function. Therefore, it will be very interesting to examine how these gain-of-function mutations affect the cellular behavior of endogenous hBest1 and the electrophysiological property of endogenous Cl^−^ in RPE^25^. From a therapeutic perspective, rather than gene supplementation for the rescue of hBest1 loss of function, a different strategy such as gene suppression may be required for the treatment of gain-of-function mutations^26^.

Although the highly conserved architecture of Best1 homologs potentiates KpBest as a simple structural model to study hBest1, one should be aware of two major limitations. First, KpBest and hBest1 share only 14% sequence identity^14^, disqualifying KpBest for the investigation of the non-conserved residues in hBest1. Second, as KpBest is a Na^+^ channel that does not require Ca^2+^ while hBest1 is a Ca^2+^-activated Cl^−^ channel^14^, KpBest is not suitable for functional studies on the Ca^2+^ dependence or ion selectivity of hBest1. In regard to these concerns, we only utilized KpBest for structural modeling of mutations on the highly conserved residues in this work.

## Methods

### Cell lines

HEK293 cells authenticated by short tandem repeat DNA profiling were kindly gifted from Dr. David Yule at the University of Rochester. HEK293 cell cultures were maintained in Dulbecco’s modified Eagle’s medium (DMEM; 4.5 g L^−1^ glucose, Corning 10013CV) supplemented with 10% fetal bovine serum and 100 μg ml^−1^ penicillin–streptomycin. 4,6-Diamidino-2-phenylindole staining revealed no mycoplasma contamination.

### Transfection

Twenty-to-24 hours before transfection, HEK293 cells were detached from cell culture dishes by treatment with 0.25% trypsin for 5 min at room temperature and thereafter split into new 3.5-cm culture dishes at 50% confluency. PolyJet transfection reagent (SignaGen SL100688) was used to transfect HEK293 cells with plasmids bearing the indicated hBest1 WT or mutant (1 μg). After 6–8 h, the transfection mix was removed and cells were washed with phosphate-buffered saline and cultured in supplemented DMEM. Twenty-four hours after transfection, cells were again detached from the cell culture dishes by trypsin and split onto fibronectin-coated glass coverslips for patch clamp^27^.

### Immunoblotting

HEK293 cells transiently expressing yellow fluorescent protein-tagged hBest1 WT and mutant channels were harvested 48 h post transfection. The M-PER Mammalian Protein Extraction Reagent (Thermo Fisher Scientific 78501) and Mem-PER Plus Membrane Protein Extraction Kit (Thermo Fisher Scientific 89842) were used to prepare the total cell lysates and membrane-associated proteins, respectively. The samples were mixed with Laemmli buffer and denatured at 95 °C for 5 min, followed by sodium dodecyl sulfate-polyacrylamide gel electrophoresis and wet transfer to nitrocellulose membranes. Immunoblotting was performed with green fluorescent protein primary antibody (1:1000, Thermo Fisher Scientific A-6455) and fluorophore-conjugated secondary antibody (1:10,000, LI-COR Biosciences 925-32213), and subsequently detected by infrared imaging.

### Electrophysiology

Electrophysiological analyses were conducted 24–72 h after transfection. An EPC10 patch clamp amplifier (HEKA Electronics) controlled by Patchmaster (HEKA)^27^ was used to perform whole-cell patch clamp recordings. Micropipettes were pulled and fashioned from filamented 1.5 mm thin-walled glass (WPI Instruments) and subsequently filled with internal solution containing (in mM): 130 CsCl, 1 MgCl_2_, 10 EGTA, 2 MgATP (added fresh), and 10 HEPES (pH 7.4, adjusted by CsOH). The desired free Ca^2+^ concentration (0 and 1.2 μM) was obtained by adding CaCl_2_ (http://maxchelator.stanford.edu/CaMgATPEGTA-TS.htm). Series resistance was typically in the range of 1.5–2.5 MΩ, with no electronic series resistance compensation. The external solution consisted of (in mM): 140 NaCl, 5 KCl, 2 CaCl_2_, 1 MgCl_2_, 10 HEPES (pH 7.4, adjusted by NaOH), and 15 glucose. When replacing Cl^−^ with CH_3_SO_3_^−^, the external solution (in mM) was 120 NaCH_3_SO_3_ and 10 HEPES (pH 7.4, adjusted by NaOH). Solution osmolarity was 310–315 mOsm. A repetition interval of 4 s was used to acquire traces^28^. Sampled at 25 kHz, currents were filtered at 5 or 10 kHz. To generate *I*–*V* curves, a group of step potentials (−100 to +100 mV from a holding potential of 0 mV) were used. The experiments were conducted at room temperature (23 ± 2 °C). Liquid junction potentials were measured and corrected using HEKA built-in functions. Solution changes were performed manually.

### Molecular cloning

All KpBest constructs have 11 residues truncated from the C-terminus of WT. Point mutations of hBest1 and KpBest were made by site-directed mutagenesis PCR with the In-fusion Cloning Kit (Clontech). All constructs were verified by sequencing.

### Protein production and purification

BL21 plysS cells were kindly gifted by Dr. Wayne Hendrickson. For protein production^15^, BL21 plysS cells containing KpBest expression vectors were cultured in Terrific Broth medium overnight, inoculated 1:100 (v/v) into fresh Terrific Broth medium, and grown at 37 °C to OD 0.6–0.8 over 2–3 h. The culture was henceforth induced with 0.4 mM IPTG and grown at 20 °C overnight.

After centrifugation at 4 °C, cell pellets were immediately processed (for purification) or stored at −80 °C until use^18^. For protein purification^29^, cell pellets were re-suspended in a buffer containing 50 mM HEPES (pH 7.8), 300 mM NaCl, 5% glycerol, 20 mM imidazole, and 0.5 mM TCEP. Cells were then lysed using an emulsiflex-C5 high pressure homogenizer (Avestin). The cell lysate was incubated with 2% DDM (w/v) for 1 h at 20 °C. The non-dissolved matter was pelleted by ultracentrifugation at 150,000 × *g* at 4 °C for 30 min. The supernatant was carefully collected and immediately loaded to a pre-equilibrated 5 mL HisTrap Ni^2+^-NTA affinity column (GE Healthcare). A 13-column volume buffer wash was performed, and the protein was eluted with a buffer containing 25 mM HEPES (pH 7.8), 200 mM NaCl, 5% glycerol, 500 mM imidazole, 0.1 mM TCEP, and 0.05% (w/v) DDM. To remove the 10×His tags, TEV proteinase was added at 1:1 mass ratio, followed by incubation at 4 °C for 30 min. The resulting samples were concentrated with 100 kDa centrifugal filter units (Amicon Ultra-15, Millipore) to a final volume of 400–500 μL. Samples were purified via size-exclusion chromatography with a Superdex-200 column by HPLC (AKTA pure 25, GE).

### Crystallization and data collection

Purified protein was concentrated to ~5 mg mL^−1^. The sitting-drop vapor diffusion method was used to grow crystals at 20 °C. The growing conditions for the best diffracted crystals are summarized in Supplementary Table 1. In all, 20% ethylene glycol was added to the crystallization solution for cryoprotection of the crystals. High-resolution native data sets from mutant KpBest crystals were collected at APS (Argonne National Laboratory) beamline 24-ID-C/E. The used X-ray wavelength was 0.979 Å, and experimental temperature was 100 K.

### Electrophysiological data and statistical analyses

Data from whole-cell patch clamp recordings were processed in Patchmaster off-line. Built-in functions in Origin were used to perform statistical analyses. According to the specific method utilized in a given experiment, we examined a sufficient number of samples to reach statistical conclusion. Student’s *t* test was used to determine statistically significant differences (*P* < 0.05) between means of two groups. Data are presented as means ± s.e.m.^30^.

The CH_3_SO_3_^−^ to Cl^−^ relative permeability (*P*_CH3SO3_/*P*_Cl_) was calculated according to the Goldman–Hodgkin–Katz equation: *ΔE*_rev_ = 59 log(150*P*_Cl_/120*P*_CH3SO3_), as the internal Cl^−^ concentration is 150 mM and the external CH_3_SO_3_^−^ is 120 mM in the presence of Ca^2+^. The relative CH_3_SO_3_^−^/Cl^−^ (with CH_3_SO_3_^−^ or Cl^−^ in the external solution) inward movement (outward current) conductance (*G*_CH3SO3 with ex-CH3SO3_/*G*_Cl with ex-Cl_) was measured as slope conductance at the reversal potential plus 50 mV, and CH_3_SO_3_^−^ current was multiplied by 1.2 to compensate for the concentration difference. The *trans* effect, representing the relative Cl^−^/Cl^−^ (with CH_3_SO_3_^−^ or Cl^−^ in the external solution) outward movement (inward current) conductance (*G*_Cl with ex-CH3SO3_/*G*_Cl with ex-Cl_) was measured as slope conductance at the reversal potential minus 50 mV.

### Structure determination and refinement

The X-ray data sets on KpBest mutants were processed and scaled using XDS^31^ via the RAPD system of APS NE-CAT and AIMLESS^32^. To solve the mutant structures, the WT KpBest structure (PDB code: 4WD8) was used as a search model during molecular replacement, which was carried out using Phaser^33^ implemented in Phenix suite^34^ and MOLREP^35^ implemented in the CCP4 suite^36^. Coot^37^, phenix.refine in the Phenix suite^34^, and Refmac5 in the CCP4 suite^38^ were utilized to carry out model building and refinement. Table 2 and Supplementary Table 2 summarizes the statistics for the diffraction data and refinement. The final models were checked by MolProbity^39^. All figures were made in PyMOL.

### Reporting summary

Further information on research design is available in the Nature Research Reporting Summary linked to this article.

## Supplementary information

Description of Additional Supplementary Files

Supplementary Information

Reporting Summary

Supplementary Data 1

## Data Availability

Data supporting the findings of this manuscript are available from the corresponding author upon reasonable request. The structures in this paper are deposited to the Protein Data Bank with access codes in Table 2 and Supplementary Table 2 (6IVR, 6IVJ, 6IVQ, 6IVM, 6IVK, 6JLF, 6IV0, 6IV1, 6IVO, 6IV2, 6IV3 and 6IV4, 6IVL, 6IVP, 6IVN, 6IVW). All source data underlying the graphs and charts presented in the main figures and primer sequences are presented in Supplementary Data 1.

## References

[1] Li, Y. et al. Patient-specific mutations impair BESTROPHIN1’s essential role in mediating Ca2^+^-dependent Cl^−^ currents in human RPE. *Elife* **6**, e29914 (2017). 10.7554/eLife.29914 PMC5655127 29063836

[2] Milenkovic A (2015). Bestrophin 1 is indispensable for volume regulation in human retinal pigment epithelium cells. Proc. Natl. Acad. Sci. USA.

[3] Moshfegh Y (2016). BESTROPHIN1 mutations cause defective chloride conductance in patient stem cell-derived RPE. Hum. Mol. Genet..

[4] Allikmets R (1999). Evaluation of the Best disease gene in patients with age-related macular degeneration and other maculopathies. Hum. Genet..

[5] Burgess R (2008). Biallelic mutation of BEST1 causes a distinct retinopathy in humans. Am. J. Hum. Genet..

[6] Davidson AE (2009). Missense mutations in a retinal pigment epithelium protein, bestrophin-1, cause retinitis pigmentosa. Am. J. Hum. Genet..

[7] Kramer F (2000). Mutations in the VMD2 gene are associated with juvenile-onset vitelliform macular dystrophy (Best disease) and adult vitelliform macular dystrophy but not age-related macular degeneration. Eur. J. Hum. Genet..

[8] Marquardt A (1998). Mutations in a novel gene, VMD2, encoding a protein of unknown properties cause juvenile-onset vitelliform macular dystrophy (Best’s disease). Hum. Mol. Genet..

[9] Petrukhin K (1998). Identification of the gene responsible for Best macular dystrophy. Nat. Genet..

[10] Yardley J (2004). Mutations of VMD2 splicing regulators cause nanophthalmos and autosomal dominant vitreoretinochoroidopathy (ADVIRC). Invest. Ophthalmol. Vis. Sci..

[11] Sun H, Tsunenari T, Yau KW, Nathans J (2002). The vitelliform macular dystrophy protein defines a new family of chloride channels. Proc. Natl. Acad. Sci. USA.

[12] Tsunenari T (2003). Structure-function analysis of the bestrophin family of anion channels. J. Biol. Chem..

[13] Kane Dickson V, Pedi L, Long SB (2014). Structure and insights into the function of a Ca(2+)-activated Cl(-) channel. Nature.

[14] Yang T (2014). Structure and selectivity in bestrophin ion channels. Science.

[15] Zhang Y (2018). ATP activates bestrophin ion channels through direct interaction. Nat. Commun..

[16] Vaisey G, Miller AN, Long SB (2016). Distinct regions that control ion selectivity and calcium-dependent activation in the bestrophin ion channel. Proc. Natl. Acad. Sci. USA.

[17] Miller AN, Vaisey G, Long SB (2019). Molecular mechanisms of gating in the calcium-activated chloride channel bestrophin. Elife.

[18] Vaisey G, Long SB (2018). An allosteric mechanism of inactivation in the calcium-dependent chloride channel BEST1. J. Gen. Physiol..

[19] Evans MG, Marty A (1986). Calcium-dependent chloride currents in isolated cells from rat lacrimal glands. J. Physiol..

[20] Frings S, Reuter D, Kleene SJ (2000). Neuronal Ca2+-activated Cl- channels--homing in on an elusive channel species. Prog. Neurobiol..

[21] Qu Z, Fischmeister R, Hartzell C (2004). Mouse bestrophin-2 is a bona fide Cl(-) channel: identification of a residue important in anion binding and conduction. J. Gen. Physiol..

[22] Xiao Q, Prussia A, Yu K, Cui YY, Hartzell HC (2008). Regulation of bestrophin Cl channels by calcium: role of the C terminus. J. Gen. Physiol..

[23] Hartzell HC, Qu Z, Yu K, Xiao Q, Chien LT (2008). Molecular physiology of bestrophins: multifunctional membrane proteins linked to best disease and other retinopathies. Physiol. Rev..

[24] Lee S (2010). Channel-mediated tonic GABA release from glia. Science.

[25] Kittredge, A., Ji, C., Zhang, Y. & Yang, T. Differentiation, maintenance, and analysis of human retinal pigment epithelium cells: a disease-in-a-dish model for BEST1 mutations. *J. Vis. Exp.*10.3791/57791 (2018). 10.3791/57791 PMC6144140 30199040

[26] Yang T, Justus S, Li Y, Tsang SH (2015). BEST1: the best target for gene and cell therapies. Mol. Ther..

[27] Yang T, He LL, Chen M, Fang K, Colecraft HM (2013). Bio-inspired voltage-dependent calcium channel blockers. Nat. Commun..

[28] Yang T, Hendrickson WA, Colecraft HM (2014). Preassociated apocalmodulin mediates Ca2+-dependent sensitization of activation and inactivation of TMEM16A/16B Ca2+-gated Cl- channels. Proc. Natl. Acad. Sci. USA.

[29] Kittredge, A., Ward, N., Hopiavuori, A., Zhang, Y. & Yang, T. Expression and purification of mammalian bestrophin ion channels. *J. Vis. Exp*. 10.3791/57832 (2018). 10.3791/57832 PMC6126599 30124653

[30] Yang T, Suhail Y, Dalton S, Kernan T, Colecraft HM (2007). Genetically encoded molecules for inducibly inactivating CaV channels. Nat. Chem. Biol..

[31] Kabsch W (2010). XDS. Acta Crystallogr. D Biol. Crystallogr..

[32] Evans PR, Murshudov GN (2013). How good are my data and what is the resolution?. Acta Crystallogr. D Biol. Crystallogr..

[33] McCoy AJ (2007). Phaser crystallographic software. J. Appl. Crystallogr..

[34] Adams PD (2010). PHENIX: a comprehensive Python-based system for macromolecular structure solution. Acta Crystallogr. D Biol. Crystallogr..

[35] Vagin A, Teplyakov A (2010). Molecular replacement with MOLREP. Acta Crystallogr. D Biol. Crystallogr..

[36] Winn MD (2011). Overview of the CCP4 suite and current developments. Acta Crystallogr. D Biol. Crystallogr..

[37] Emsley P, Cowtan K (2004). Coot: model-building tools for molecular graphics. Acta Crystallogr. D Biol. Crystallogr..

[38] Murshudov GN (2011). REFMAC5 for the refinement of macromolecular crystal structures. Acta Crystallogr. D Biol. Crystallogr..

[39] Chen, V. B. et al. MolProbity: all-atom structure validation for macromolecular crystallography. *Acta Crystallogr. D Biol. Crystallogr. ***66**, 12–21 (2010). 10.1107/S0907444909042073 PMC2803126 20057044

