# Supplementary material for: Dual Ca2+-dependent gates in human Bestrophin1 underlie disease-causing mechanisms of gain-of-function mutations
Source: Commun Biol. 2019 Jun 24;2:240. doi: 10.1038/s42003-019-0433-3 (PMC6591409; doi:10.1038/s42003-019-0433-3)
Supplement: Supplementary file 1 — Description of Additional Supplementary Files [file 42003_2019_433_MOESM1_ESM.docx]

**Description of additional supplementary items**

**Data File name:** Supplementary Data 1

**Description:** The Supplementary Data 1 file contains all source data underlying the graphs and charts presented in the main figures (Figure 1-7).
