# Supplementary material for: Dual Ca2+-dependent gates in human Bestrophin1 underlie disease-causing mechanisms of gain-of-function mutations
Source: Commun Biol. 2019 Jun 24;2:240. doi: 10.1038/s42003-019-0433-3 (PMC6591409; doi:10.1038/s42003-019-0433-3)
Supplement: Supplementary file 2 — Supplementary Information [file 42003_2019_433_MOESM2_ESM.pdf]

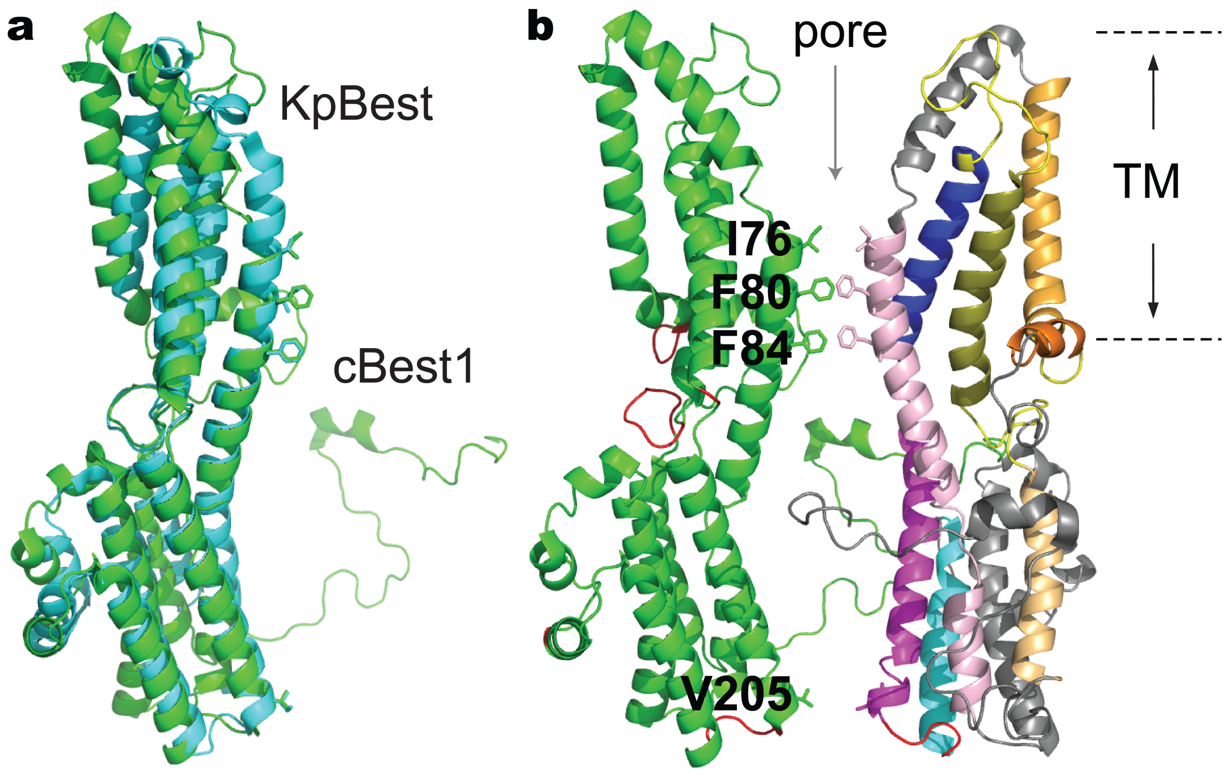

Supplementary Figure 1. Structural analysis of bestrophin channels. (**a**) Structure alignment of KpBest (blue) and cBest1 (green) as shown by superposition of their protomers. (**b**) Ribbon diagram of two oppositely facing (144°) protomers of a cBest1 pentamer is shown with the extracellular side on the top. The loops residing the conserved residues subjected for alanine substitution screen are labeled in red on the left protomer, while the surrounding helices are labeled in the same colors as those in Figs. 4-7 on the right protomer.

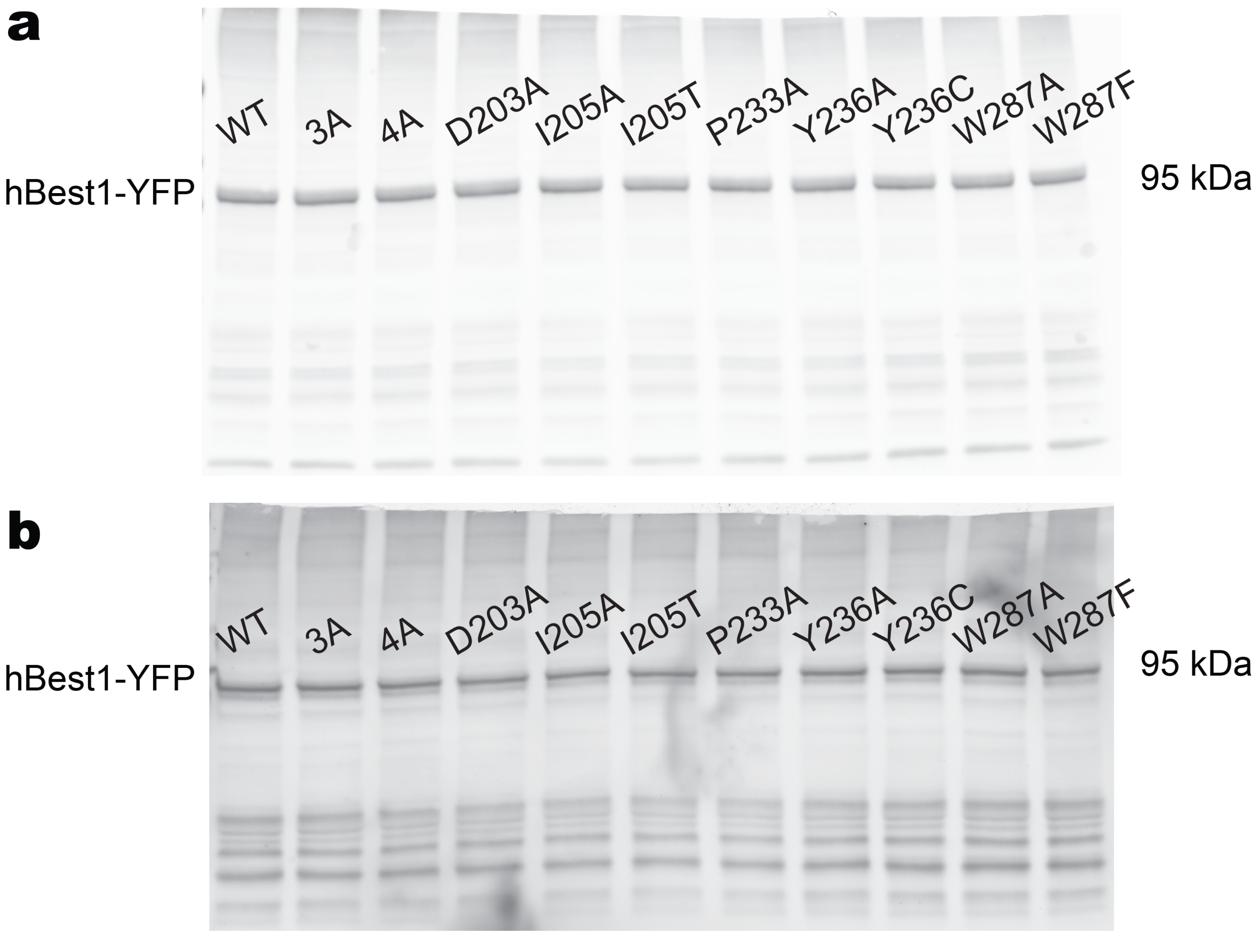

Supplementary Figure 2. Expression and membrane trafficking of hBest1 mutants. (**a-b**) Western blot showing the expression levels of hBest1 WT and mutants in whole-cell lysate (**a**) or membrane fraction (**b**) of transfected HEK293 cells.

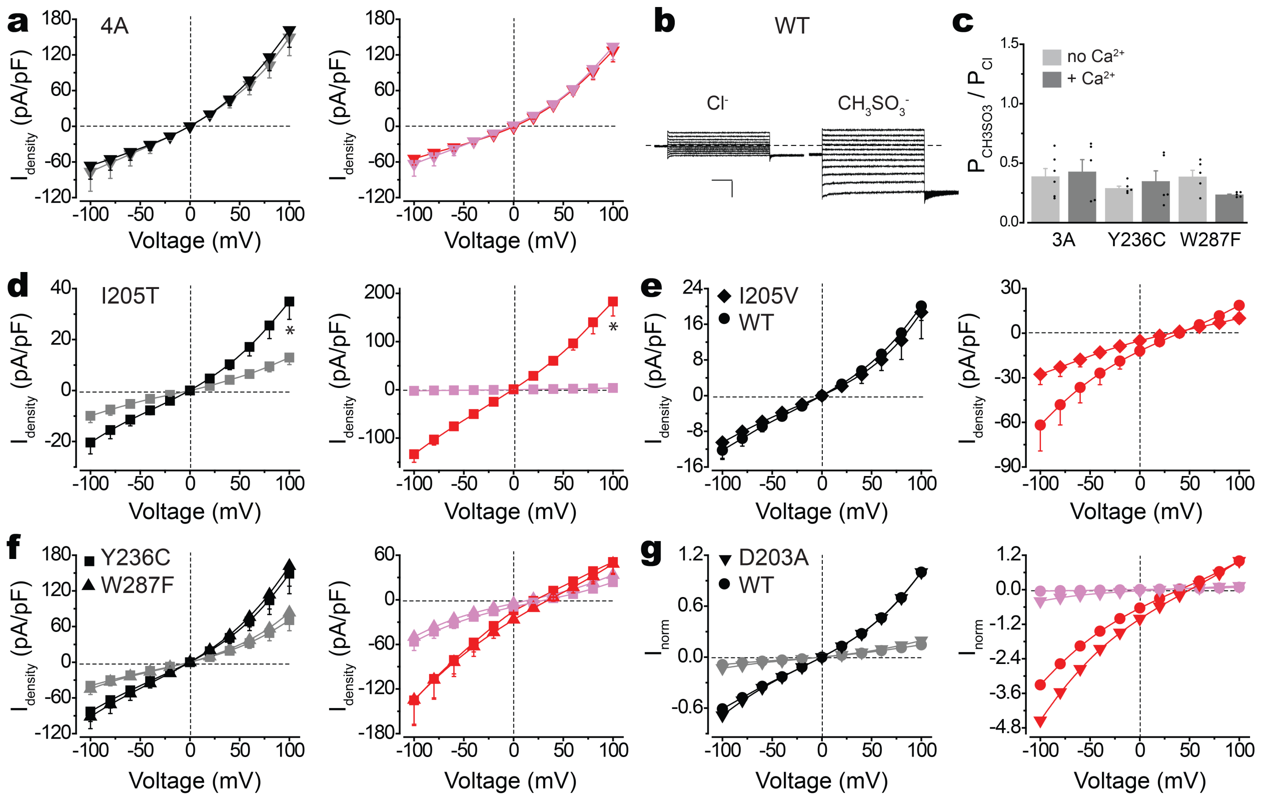

Supplementary Figure 3. Ca^{2+}-dependent anion currents in hBest1 WT and mutants. (**a**) Population steady-state current density-voltage relationships in HEK293 cells expressing hBest1 4A, with Cl^- in the external solution, in the absence (gray) or presence (black) of 1.2 μM Ca^{2+}, or with $CH_3SO_3^-$ in the external solution in the absence (magenta) or presence (red) of 1.2 μM Ca^{2+}, n= 7-12 for each point. (**b**) Representative current traces from WT hBest1 in the presence of 1.2 μM Ca^{2+}, with Cl^- or $CH_3SO_3^-$ in the external solution. Scale bar, 300 pA, 150 ms. (**c**) Bar chart showing the relative ion permeability ratios (P_{CH3SO3}/P_{Cl}) of hBest1 3A, Y236C and W287F, calculated from the Goldman-Hodgkin-Katz equation in the absence or presence of Ca^{2+}, n= 5-6 for each bar. (**d**) Population steady-state current density-voltage relationships in HEK293 cells expressing hBest1 I205T, with Cl^- in the external solution, in the absence (gray) or presence (black) of 1.2 μM Ca^{2+}, or with $CH_3SO_3^-$ in the external solution in the absence (magenta) or presence (red) of 1.2 μM Ca^{2+}, n= 10-11 for each point. $^*P < 0.05$ compared to cells in the absence of Ca^{2+}, using two-tailed unpaired Student's *t* test. (**e**) Population steady-state current density-voltage relationships in HEK293 cells individually expressing hBest1 WT and I205V with Cl^- (black) or $CH_3SO_3^-$ (red) in the external solution, in the presence of 1.2 μM Ca^{2+}, n= 5-6 for each point. (**f**) Population steady-state current density-voltage relationships in HEK293 cells individually expressing hBest1 Y236C and W287F, with Cl^- in the external solution, in the absence (gray) or presence (black) of 1.2 μM Ca^{2+}, or with $CH_3SO_3^-$ in the external solution in the absence (magenta) or presence (red) of 1.2 μM Ca^{2+}, n= 5-11 for each point. (**g**) Normalized population steady-state mean current density-voltage relationships in HEK293 cells individually expressing hBest1 WT and D203A, with Cl^- in the external solution, in the absence (gray) or presence (black) of 1.2 μM Ca^{2+}, or with $CH_3SO_3^-$ in the external solution in the absence (magenta) or presence (red) of 1.2 μM Ca^{2+}, n= 8-14 for each point. All error bars in this figure represent s.e.m.

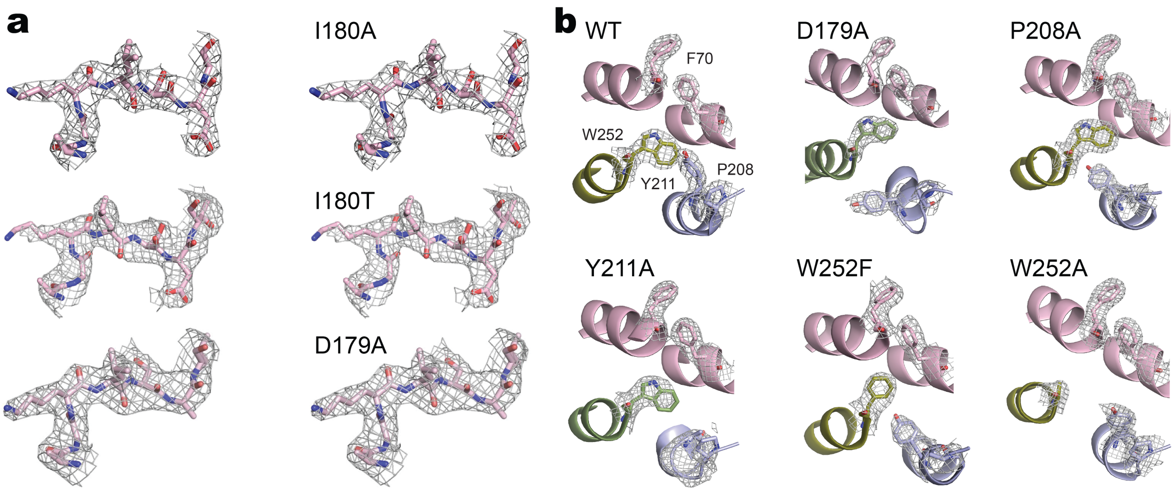

Supplementary Figure 4. Crystal structures of KpBest mutants. (**a**) Stereo images of the electron density maps (2Fo-Fc map, 1.2σ level) of KpBest I180A, I180T and D179A, presenting residues 174 - 180 for divergent "wall-eyed" viewing. (**b**) Visualization of the neck region of KpBest WT and mutants, showing electron density maps (2Fo-Fc map, 1.2σ level) of critical residues (F70, P208, Y211 and W252). Helices surrounding the critical residues are labeled in the same colors as those in Figs. 4-6 for comparison.

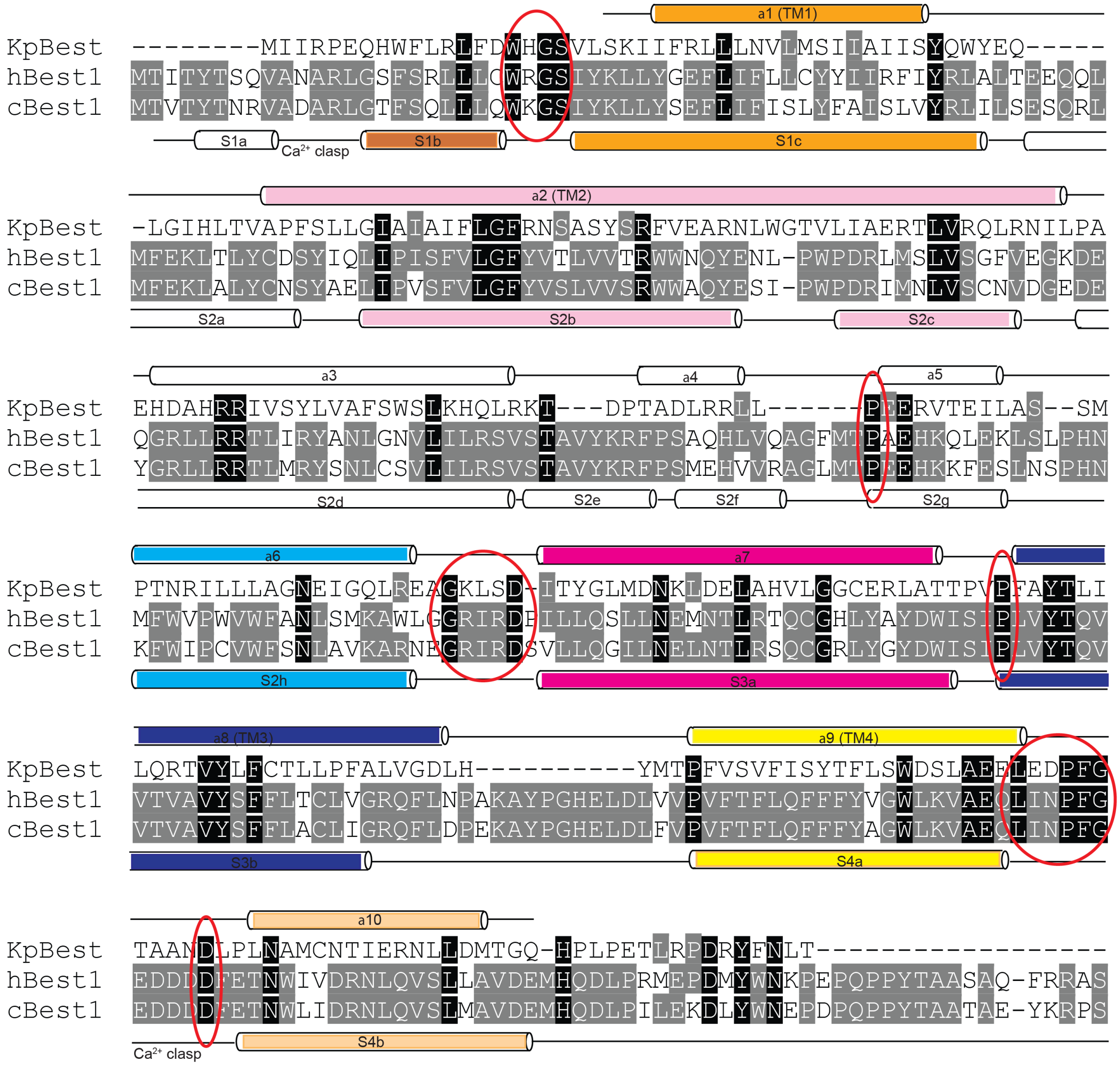

Supplementary Figure 5. Structure-based sequence alignment of KpBest, hBest1 and cBest1. The KpBest structure is used to restrict sequence gaps to inter-helical segments. Black background, identical residues in all three species; gray background, identical residues in two species. The secondary structures of KpBest and cBest1 are labeled above and below the sequences, respectively. The loops residing alanine substituted residues are circled in red. Critical helices potentially involved in channel activation are highlighted in the same colors as those in Supplementary Fig. 1.

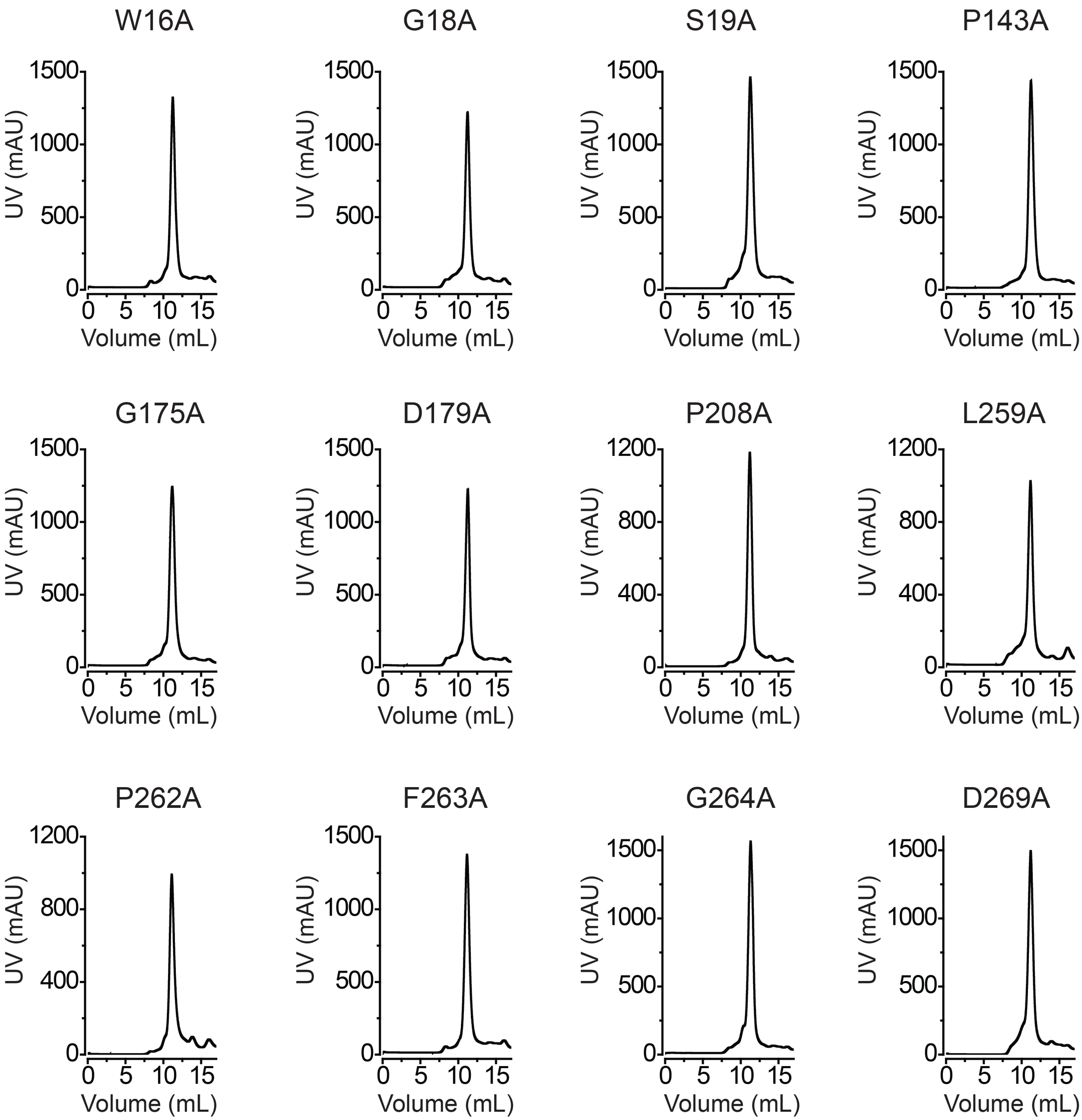

Supplementary Figure 6. The elution profiles of purified KpBest loop mutants. Representative size exclusion profile of purified KpBest mutant proteins.

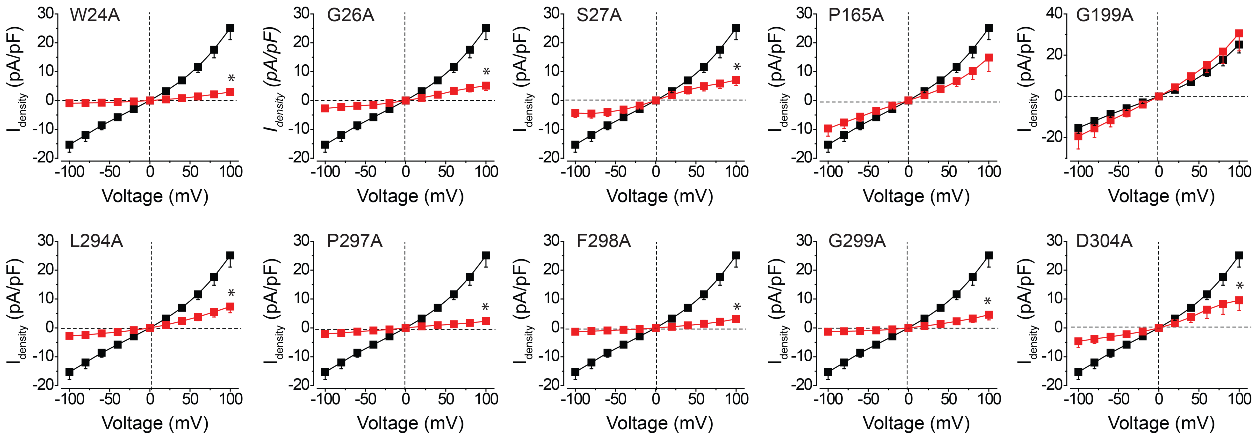

Supplementary Figure 7. Functional analysis of hBest1 loop mutants. Population steady-state current density-voltage relationships in HEK293 cells expressing WT hBest1 (black) or loop mutants (red), with Cl^- in the external solution and in the presence of 1.2 μM Ca^{2+}, n= 5-10 for each point. *P < 0.05 compared to WT hBest1, using two-tailed unpaired Student's *t* test. All error bars in this figure represent s.e.m.

Supplementary Table 1 Mutations in hBest1/KpBest (1-12 are loop mutations)

#	Residue in hBest1	Position in KpBest/cBest1	Corresponding residue in KpBest	Mutation made	COOT SSM Superpose to WT (RMSD, Å)	Conditions
1	W24	Loop	W16	A	0.3	ZnAce 0.05 M, PEG 8000 6.6%, NaCacodylate 0.1 M (pH 6.0), EG 6 %
2	G26	Loop	G18	A	0.4	ZnAce 0.05 M, PEG 8000 6.6%, NaCacodylate 0.1 M (pH 6.0), EG 6 %
3	S27	Loop	S19	A	0.4	ZnAce 0.03 M, PEG 8000 6.6%, NaCacodylate 0.1 M (pH 6.0), EG 6 %
4	P165	Loop	P143	A	0.4	ZnAce 0.05 M, PEG 8000 6.6%, NaCacodylate 0.1 M (pH 6.0), EG 6 %
5	G199	Loop	G175	A	0.4	ZnAce 0.03 M, PEG 8000 6.6%, NaCacodylate 0.1 M (pH 6.0), EG 6 %
6	D203	Loop	D179	A	1.0	ZnAce 0.05 M, PEG 8000 7.0%, NaCacodylate 0.1 M (pH 5.0), EG 6 %
7	P233	Loop	P208	A	0.6	ZnAce 0.01 M, PEG 8000 6.6%, NaCacodylate 0.1 M (pH 6.0), EG 6 %
8	L294	Loop, Ca^{2+} clasp	L259	A	0.7	ZnAce 0.05 M, PEG 8000 6.6%, NaCacodylate 0.1 M (pH 6.0), EG 6 %
9	P297	Loop, Ca^{2+} clasp	P262	A	0.5	ZnAce 0.05 M, PEG 8000 7.5%, NaCacodylate 0.1 M (pH 5.0), EG 6 %
10	F298	Loop, Ca^{2+} clasp	F263	A	NA	NA
11	G299	Loop, Ca^{2+} clasp	G264	A	0.5	ZnAce 0.05 M, PEG 8000 6.6%, NaCacodylate 0.1 M (pH 5.5), EG 6 %
12	D304	Loop, Ca^{2+} clasp	D269	A	1.0	ZnAce 0.10 M, PEG 8000 9.0%, NaCacodylate 0.1 M (pH 6.0), EG 6 %
13	I205	Aperture	I180	A	0.4	ZnAce 0.05 M, PEG 8000 6.6%, NaCacodylate 0.1 M (pH 6.0), EG 6 %
14	I205	Aperture	I180	T	0.5	ZnAce 0.05 M, PEG 8000 6.6%, NaCacodylate 0.1 M (pH 6.0), EG 6 %
15	Y236	Helix	Y211	A	0.8	ZnAce 0.05 M, PEG 8000 7.0%, NaCacodylate 0.1 M (pH 6.0), EG 6 %
16	W287	Helix	W252	A	1.0	ZnAce 0.01 M, PEG 8000 6.6%, NaCacodylate 0.1 M (pH 6.0), EG 6 %
17	W287	Helix	W252	F	1.2	ZnAce 0.05 M, PEG 8000 7.0%, NaCacodylate 0.1 M (pH 6.0), EG 6 %

Supplementary Table 2 X-ray data refinement statistics

	W16A	G18A	S19A	P143A	G175A	D179A	I180A	I180T	P208A	Y211A	W252A	W252F	L259A	P262A	G264A	D269A
**Ramachandran plot**																
PDB code ID	6IVR	6IVJ	6IVQ	6IVM	6IVK	6JLF	6IV0	6IV1	6IVO	6IV2	6IV3	6IV4	6IVL	6IVP	6IVN	6IVW
Favored (%)	95.29	93.26	96.11	95.96	95.14	97.28	93.48	96.24	97.69	96.83	97.25	90.99	90.26	90.74	97.76	92.31
Allowed (%)	4.19	6.44	3.07	3.22	4.11	2.49	6.07	3.54	1.79	2.87	2.15	7.19	9.29	8.51	2.02	7.08
Disallowed (%)	0.52	0.30	0.82	0.82	0.75	0.23	0.45	0.23	0.52	0.3	0.59	1.82	0.45	0.75	0.22	0.6
